# The Global Impact of COVID‐19 Control Measures on People With Dementia Living at Home and Their Carers: A Systematic Review of Quantitative and Qualitative Research Across 27 Countries

**DOI:** 10.1002/brb3.71100

**Published:** 2025-11-24

**Authors:** Yaohua Chen, Tatyana Mollayeva, Rachael Fitzpatrick, Thaisa Tylinski Sant'Ana, Francesca Farina, Dorota Swiatek, Kelli Sopidou, Evelyn Tabilo, Marta Betka, Iracema Leroi, Tomas Leon, Geeske Peeters, the STRAP consortium

**Affiliations:** ^1^ Department of Geriatrics Univeristy of Lille, CHU Lille, U1172, LilNCog Lille France; ^2^ Global Brain Health Institute Trinity College Dublin, University of California San Francisco, Dublin San Francisco; ^3^ KITE‐Toronto Rehab‐University Health Network Toronto Ontario Canada; ^4^ Dalla Lana School of Public Health University of Toronto Toronto Ontario Canada; ^5^ Department of Psychiatry, School of Medicine Trinity College Dublin Dublin Ireland; ^6^ Pritzker School of Medicine University of Chicago Chicago Illinois USA; ^7^ Regional Specialist Hospital in Wrocław Research and Development Center Wroclaw Poland; ^8^ Neurosciences and Neurodegenerative diseases, Medical School, Faculty of HealthSciences Aristotle University Athens Greece; ^9^ Gerosciences Center for Brain Health and Metabolism (GERO) Santiago Chile; ^10^ ⁠Memory and Neuropsychiatric Center (CMYN) Neurology Department, Hospital del Salvador & Faculty of Medicine University of Chile Santiago Chile; ^11^ Mazowiecki Szpital Bródnowski Warsaw Medical University Warsaw Poland; ^12^ Department of Psychiatry, School of Medicine Trinity College Dublin Dublin Ireland; ^13^ Radboudumc Alzheimer Center and Department of Geriatric Medicine Radboud University Medical Center Nijmegen the Netherlands

**Keywords:** carers, dementia, healthcare access, mental health, prognosis, SARS‐CoV‐2

## Abstract

**Background:**

COVID‐19 control measures have had a unique impact on people with dementia (PWD) and their carers living at home. Yet, uncertainty exists regarding the global impact of such measures and whether differences exist between countries and global regions. We aimed to synthesize evidence on this topic.

**Methods:**

We searched Medline, PsycINFO, EMBASE, Web of Science, CINAHL, Latin American and Caribbean Health Literature (LILACS), Scientific Electronic Library Online (SciELO), and EM Premium from the start of the pandemic to July 2022. At least two researchers independently screened citations and performed quality assessment following recommended criteria for critical appraisal according to study methodology. We analyzed data by country and region and synthesized results descriptively.

**Results:**

Sixty‐nine studies met inclusion criteria (74% quantitative and 26% qualitative; 22% included PWD, 44% carers of PWD, and 4% dyads), with a total of 209,738 participants. Most studies were conducted in Europe (59%), followed by Asia and North America (15% each), South America (7%), and Oceania (1%). Two studies presented data from multiple regions (3%). The quality of the studies varied, with the majority (62%) being of moderate quality. Across the study populations and global regions, COVID‐19 control measures had implications for PWD and carers’ access to health services, physical and mental health and daily routine, cognition, behavior, with accompanying social and economic costs. The impact on mental health for PWD and on loneliness and well‐being for carers were the two most frequently studied outcomes.

**Conclusion:**

People with dementia and their carers represent a heterogeneous group of people across countries and communities; despite that, the impacts of COVID‐19 control measures on PWD and their carers were broadly consistent across regions. Our evidence synthesis highlights the critical need for decision‐makers to account for the needs of PWD and their carers when designing and implementing public health measures.

**Other:**

This work was funded by the JPND Call for Expert Working Groups: The Impact of COVID‐19 on Neurodegenerative Diseases in partnership with the CIHR‐Institute of Aging and the Public Health Agency (CIHR #02342‐000). PROSPERO CRD42024554701.

## Graphical abstract

This graphical abstract summarizes the main findings of our systematic review about the global impacts of COVID‐19 control measures on people with dementia living at home and their carers : they were broadly consistent across regions. Our evidence synthesis highlights the critical need for decision‐makers to account for the needs of PWD and their carers when designing and implementing public health measures.

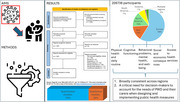

**Abbreviations**
AD: Alzheimer's disease
COVID‐19: Coronavirus disease
DEMQOL: Dementia Quality of Life
DEMQOL‐C: Dementia Quality of Life of Carers
EQ‐5D: EuroQol Five‐Dimensions
FTD: frontotemporal dementia
MMSE: Mini‐Mental State Examination
PRISMA: Preferred Reporting Items for Systematic Reviews and Meta‐Analyses
PRISMA: Preferred Reporting Items for Systematic reviews and Meta‐Analyses
PROSPERO: International prospective register of systematic reviews
PWD: Persons with dementia
QUALID: Quality of Life in Late‐stage Dementia
SARS‐CoV‐2: severe acute respiratory syndrome coronavirus 2
SF‐12: 12‐item Short Form Survey
SWEMWBS: Short Warwick‐Edinburgh Mental Wellbeing Scale
USA: United States of America
WAIS: Wechsler Adult Intelligence Scale

## 1 Introduction

The COVID‐19 pandemic and its control measures have had a significant impact on both the physical and mental health of communities across the globe, with up to 50% of the general population reporting increased psychological distress, depression, and reduced quality of life (Lee et al. 2021; Oluwasegun Ayenigbara, 2022). People with dementia (PWD), including those living at home during the pandemic, were reported to be affected by the lockdowns, physical distancing, and other containment strategies across healthcare systems globally (Magklara and Kyriakopoulos, 2023; Bianchetti et al. 2022; Barguilla et al. 2020; Bakker et al. 2022).

It has become apparent that the global response to the pandemic had implications for both PWD and their carers. Research suggests that control measures increased the cognitive and/or physical decline in PWD (Kennedy et al. 2024; Grycuk et al. 2022; Vislapuu et al. 2021) and impacted the health and well‐being of carers of PWD (Barguilla et al. 2020), who had to take on care responsibilities previously held by formal care while managing their own stress, anxiety, and other mental health concerns. These implications may be relevant across dementia types and disease stages, affecting PWD and their carers of all ages, sexes, gender identities, ethnicities, and places of residence (Canevelli et al. 2020; Hanafy et al. 2023; Suárez‐González et al. 2021; Livingston et al. 2020). As such, the impact of COVID‐19 control measures on PWD and their carers living at home during the pandemic requires comprehensive evaluation.

To date, several studies have systematically reviewed data on the impact of control measures on PWD, predominantly those of quantitative methodologies focusing largely on PWD living in care facilities during the pandemic (Bicalho et al. 2024; Tavares‐Júnior et al. 2022; Gaigher et al. 2022). The results suggested decreased infection rates and increased survivorship as a result of control measures (Hariyanto et al. 2021; Talic et al. 2021; Burns et al. 2021; Stratil et al. 2021). However, in light of emerging qualitative evidence on the implications of the lockdown on mental health and well‐being, there is a need to integrate both qualitative and quantitative evidence on the impact of the COVID‐19 control measures on PWD living at home and their carers through a global lens. This is particularly important because COVID‐19 control measures varied widely across countries and regions (Salanti et al. 2022; Wang et al. 2021; Alkhaldi et al. 2021), reflecting differing public health policies, healthcare infrastructures, and pandemic severities. This variability may have led to different impacts on PWD living in these countries, which requires further data synthesis to understand the global scope and identify patterns, gaps, and best practices that may inform future responses to similar crises.

Therefore, we conducted a systematic review of qualitative and quantitative evidence with the following aims: (1) to assess the impact of pandemic‐related control measures on PWD living at home and their carers; (2) to compare results across countries and regions to identify commonalities and differences; and (3) to offer an evidence‐based discussion for policymakers, healthcare providers, and support organizations to improve care for PWD during future emergencies.

## 2 Methods

We registered the review with the International Prospective Register of Systematic Reviews (PROSPERO, CRD42024554701). We conducted data extraction, analyses, and reporting in compliance with the Preferred Reporting Items for Systematic Reviews and Meta‐Analyses (PRISMA) guidelines (Page et al. 2021) (Supplementary Material 1).

### 2.1 Data Source and Searches

We identified all peer‐reviewed studies, irrespective of the country or study setting, published from March 11, 2020, to the last day of July 2022 through Medline, PsycINFO, EMBASE, Web of Science, CINAHL, Latin American and Caribbean Health Literature (LILACS), Scientific Electronic Library Online (SciELO), and EM Premium. The medical subject headings (MeSH) for Medline were “dementia,” “mental health,” “COVID‐19,” and “care partners.” We also searched grey literature using Google Scholar (the first 10 pages) and an inventory of existing national surveys and reports in each participant's country for items published from the start of the pandemic through the end of July 2022. We cross‐checked the references list of all included studies.

### 2.2 Eligibility Criteria Based on the PICOS Approach for Quantitative Studies

**P** (Participants): people with dementia and/or their carers living at home/community dwelling during the COVID‐19 pandemic;
**I** (Interventions): not applicable;
**C** (Comparisons): not applicable;
**O** (Outcomes): disease progression, physical/cognitive/daily functioning, mental health, well‐being, access to care/health services, carer burden, or social outcomes in the context of COVID‐19 control measures;
**S** (Study design): quantitative observational studies of any design and qualitative studies of lived experiences of dementia and/or their carers living at home.

### 2.3 Eligibility Criteria Based on the PICoS Approach for Qualitative Studies

‐ P (Population): People with dementia and their informal/family carers living at home during the COVID‐19 pandemic.
‐ I (Phenomenon of Interest): Experiences and perceived impacts of COVID‐19 control measures (e.g., lockdowns, isolation, healthcare disruption).
‐ Co (Context): Home‐ or community‐based settings in various global regions.
‐ S (Study design): Qualitative studies using interviews, focus groups, or ethnographic approaches.

### 2.4 Inclusion and Exclusion Criteria

We considered original peer‐reviewed observational or qualitative studies in any language of all designs that (1) included people with dementia and/or their carers living at home/community dwelling during COVID‐19 and (2) assessed their physical/cognitive/daily functioning, mental health, well‐being, access to care/health services, or other health, social, and/or functional outcomes. In addition, the studies were required to have (3) quantified the magnitude of the association between COVID‐19 control measures and the outcome of interest or reported on the implications of COVID‐19 control measures on PWD and/or their carers living at home.

We excluded non‐human studies, letters to editors, reviews, conference abstracts, articles without primary data, single case studies, studies that focused on therapeutic interventions, and studies that included people living in long‐term care facilities or nursing homes and whose samples were composed largely of people with a diagnosis of mild cognitive impairment or other disorders of a neurodegenerative nature. We also excluded studies that focused on investigating the effect of the COVID‐19 infection itself on PWD and/or their carers.

### 2.5 Data Collection and Analysis

#### 2.5.1 Selection Process

We extracted keyword‐based searches into an online reference management tool (EndNote, version 20, Clarivate, Philadelphia, PA, USA). One researcher (Y.C.) eliminated duplicates and inserted data into a collaborative online tool (Covidence, Melbourne, Australia). Two researchers per title (D. S., K. S., E. T., M. B., Z. A., Y. C., T. L., R. F.) independently assessed the study titles and abstracts for compliance with the inclusion criteria. Differences in opinion were resolved through group discussion. Then, each researcher assessed the selected full texts to determine their compliance with inclusion/exclusion criteria. When information regarding any of the above was unclear, we contacted authors of the reports to provide further details.

#### 2.5.2 Data Extraction

Two researchers per study (D. S., K. S., E. T., M. B., Y. C., T. L., G. P., R. F., F. R. F.) independently extracted the data into Excel files, consisting of (1) study information (authors, publication year, country, objectives, design, method of recruitment, sample size, measures used, and effects of COVID‐19 control measures investigated in the study) and (2) participant information (age, sex, dementia type and severity if PWD, and relationship to the participant if care partners). Outcomes were categorized into the following themes: physical/daily functioning; cognitive symptoms; mental health; behavior; well‐being/loneliness; social cost; access to care; and other. We further collapsed the categories into themes based on conceptual unity emerging from data synthesis: (1) physical health and daily routine; (2) cognitive functioning; (3) behavioral problems, mental health and well‐being; (4) social and economic consequences; and (5) access to health services.

### 2.6 Risk of Bias and Quality Assessment

Four researchers (Y. C., I. L., R. F., T. M.) independently assessed study quality using the National Institutes of Health study quality assessment tools (National Institutes of Health 2013) that suited each included study design. The researchers involved in the assessment of risk of bias initially met for a calibration review, in which they independently reviewed one study of each type and discussed each item on the list to clarify its meaning and interpretation. Following this, the same three reviewers independently rated the methodological quality of each study across a set of items. In cases of disagreement between the researchers, a team discussion took place, with the aim to reach consensus.

#### 2.6.1 Quantitative Studies

We appraised quantitative studies in two steps. The first step included rating items related to potential sources of bias according to the most critical criteria for external and internal validity within cohort, cross‐sectional, or case‐control studies. The second step consisted of summarizing the presence of potential sources of biases as “yes,” “no,” “not reported,” or “cannot determine.” The overall rating of potential bias for each study was summarized in accordance with the Scottish Intercollegiate Guidelines Network methodology (Scottish Intercollegiate Guidelines Network 2011), which summarized study quality into three groups: high‐quality (“++”), when all or most of the quality was fulfilled (i.e., allowing one “CD” or “NR” based on six potential sources of bias); moderate‐quality (“+”), when half of the items of criteria were fulfilled; or low‐quality (“−”) when less than half of the items of criteria were fulfilled (Supplementary Material 3).

#### 2.6.2 Qualitative Studies

We evaluated qualitative studies using the Critical Appraisal Skills Programme (CASP) qualitative studies checklist (Critical Appraisal Skills Programme 2024). Assessments were categorized as follows: “Yes” indicated that the item was adequately addressed in the study, “no” indicated that the item was not clearly addressed, and “cannot tell” reflected uncertainty. A score was given for each item that was categorized as “yes.” Study quality was then summarized into three groups: high‐quality (“++”), when all or most of the criteria were fulfilled; moderate‐quality (“+”), when half of the items of the criteria were fulfilled; or low‐quality (“−”), when less than half of the criteria were fulfilled (Supplementary Material 2).

### 2.7 Data Synthesis

We tabulated the study characteristics into a file to evaluate similarities between studies. We observed heterogeneity across all PICOS criteria, confirming that the assumptions for conducting a classical meta‐analysis were not met. Further, variations in outcome definition and categorization, and differences in the study samples and characteristics prevented us from performing data conversions. We therefore used a best‐evidence synthesis approach, synthesizing findings from included studies through tabulation, data visualization, and qualitative description (Slavin, 1995; Ayorinde et al. 2020). We summarized the evidence and presented an overview of the findings across the studied outcome categories according to quantitative and qualitative data by global region.

### 2.8 Sensitivity Analysis

We carried out sensitivity analysis to test whether critical methodological concerns affected the results. This involved repeating an analysis using combined studies regardless of study quality and separately and informally comparing the findings. We grouped the main results by study design (quantitative and qualitative) and compared the results of studies that focused on the same outcome theme.

### 2.9 Publication Bias

Due to high heterogeneity of study design, population, COVID‐19 waves, and outcomes (Table 1), and variability among studies in the bias domains affecting study quality (Supplementary Materials 2 and 3), we did not perform evaluation of the publication bias using statistical tests (Ayorinde et al. 2020). To minimize the potential for such bias, we implemented several strategies, including conducting literature searches with attempts to locate grey literature and unpublished studies and assessing reporting bias in outcomes of included studies (Dwan et al. 2013).

**TABLE 1:** Descriptive characteristics of included studies (1/7).

								People with d*ementia*			*Carers*		
Author (Year)	Country	Continent	Study design	Start date–End date	COVID wave	*N*	Participants	Sex % female	Age mean (SD)^a^	Stage %	Sex % female	Age mean (SD)^a^	Relationship to caree (%)
Alexopoulos (2021)	Greece	Europe	CSS			67	Carers: 100%	70.10%	80.2 (7.7)	mild: 28.4% mod: 43.3% severe: 9.0%	82%	58.3 (12.2)	spouse: 29.9% child: 64.2%
Altieri (2021)	Italy	Europe	CSS	April 21–May 3 2020		84	Carers: 100%	72.60%	78.5 (10.1)		84.50%	48.7 (11.7)	spouse: 11.9% child: 72.6%
Azevedo (2021)	Argentina Brazil Chile	South America	CSS	May–July 2020		321	Carers: 100%	57%	77.2 (9.4)	mild: 50.2% mod: 23.1% severe: 26.8%	78.80%	58.8 (15.3)	
Bakker (2022)	Netherlands	Europe	CSS	December 2020–March 22 2021	2	1337	Carers: 61.8% PWD: 8.9% SCD: 23%	39.90%	67 (8)		61%	67 (11)	
Bannon (2021)	USA	North America	QI	March–August 2020		46	PWD‐carer dyads: 100%	57%	61.3 (4.7)		48%	60.5 (5.4)	spouse: 100%
Barguilla (2020)	Spain	Europe	CSS	March–May 2020	1	60	PWD: 75% MCI: 25%	53.3	75.4 (5.2)	mild: 44% mod: 36% severe: 20%			
Baumbusch (2022)	Canada	North America	QI	August 2020–August 2021	2, 3	12	Carers: 100%	50%	82 (range 62‐101)		83%	61.8 (range 36‐82)	spouse: 41.7% child: 41.7% sibling: 8.3%
Boutoleau‐Bretonniere (2020)	France	Europe	CSS	March 26–May 9 2020		76	Carers: 100%	AD: 60.5% FTD: 42.1%	AD: 71.9 (8.2) FTD: 67.8 (9.0)		60.10%	64.2	spouse: AD: 78.9% FTD: 89.5%
Busse (2022)	Italy	Europe	PR	March 2020–April 2021		135	Carers: 63% Non‐carers: 37%		74.6 (113)	mild: 60% mod: 19% severe: 21%	carers: 69.4% non‐carers: 64%	carers: 62 (14.6) non‐carers: 62 (11.2)	spouse: 57.6%
Chen (2021)	China	Asia	PR	September 30 2019–November 31 2020		177	PWD: 71.5% MCI: 28.5%	AD 58% DLB 45.4%	AD: 71.5 (8.1) DLB: 74.0 (7.9)	mild: 41.1% mod: 33.4% severe: 25.6%			
Cipolletta (2021)	Italy	Europe	QI	April 8—May 2 2020		20	Carers: 100%	65%			85%	53 (range 42‐67)	child: 100%
Table 1 Descriptive characteristics of included studies (2/7).													
Cohen (2020a)	Argentina	South America	CSS	May 2020	1	119	Carers: 100%	64.70%	81.2 (7.0)	mild: 34.5% mod: 32% severe: 33%	71.90%	58.6 (13.6)	
Cohen (2020b)	Argentina	South America	CSS	April 2020	1	80	Carers: 100%	62.50%	80.5 (7.7)	mild: 28.8% mod: 41.3% severe: 30%	69.20%	56.2 (14.1	
Daley (2022)	UK	Europe	PR	Mar 2018–Mar 2020	1, 2	248	Carers: 100%	41.50%	77.5 (8.0)	mild: 42.7% mod: 29.4% severe: 4.4%	68.10%	70.1 (10.6)	spouse: 79.4% child: 19.8%
Flemons (2022)	Canada	North America	QI	June–July 2020	1	21	Carers: 100%				100%	63	spouse: 40% child: 55% sibbling: 5%
Gamble (2022)	UK	Europe	PR	September 21 2020–April 30 2021	1, 2	468	Carers: 100%				BL: 63.1% DL: 62.8%	69	spouse: 89.7%
Gan (2021)	China	Asia	PR	DL: January 1 2019–November 30 2020 BL: January 2017 ‐ December 2018		205	PWD: 100%	50.2%	70.6 8.0)				
Geyer (2020)	Germany	Europe	QI	April–May 2020		21	Carers: 85.7% PWD: 14.3%			mild‐mod: 100%			spouse: 77.8% child: 22.2%
Giebel (2020)	UK	Europe	CSS	April–May 2020		569	Healthy older adults: 39.2% carers: 50.1% PWD: 10.7%	44.30%	70 (10)		77.10%	61+‐13	
Giebel (2020)	UK	Europe	QI	April–May 2020		15	Carers: 100%				93.30%	59.6 (7.2)	spouse: 53.3% child: 46.7%
Giebel (2021)	UK	Europe	QI	April–July 2020		50	Carers: 84% PWD: 16%	37.50%			83.30%		spouse: 55%
Giebel (2021)	UK	Europe	QI	April 2020	1	50	Carers: 84% PWD: 16%					60 (9)	spouse: 55%
Table 1 Descriptive characteristics of included studies (3/7).													
Giebel (2021)	UK	Europe	PR	April–August 2020	1	569	Healthy older adults: 39.2% Carers: 50.1% PWD: 10.7%	44.3%	70 (10)		77.1%	61 (13)	
Grycuk (2022)	Ireland New Zealand UK USA	Europe/Oceania/North America	CSS	June–November 2020		2287	Carers: 100%				81.70%	45+ yrs: 90.3%	
Hashimoto (2020)	Japan	Asia	PR	April 8–April 28 2020		111	Healthy older adults: 33.3% PWD: 66.7%	63.50%	Living alone: 80.9 (7.9) Living together: 75.4 (6.3)				
Helvaci Yilmaz (2021)	Turkey	Europe	CSS			54	PWD: 100%		77.2 (6.9)		63%		spouse: 14.8% child: 72.2%
Hicks (2022)	UK	Europe	PR	July–October 2020		207	PWD: 100%		80.3 (8.25)	mild: 72% mod: 25% severe: 2%		66 (13.8)	
Ismail (2021)	Kuwait	Asia	CSS	September 2020		36	PWD: 100%	63.90%	71 (10.8)	BL: mild: 36.1% mod: 47.2% severe: 8.3% DL: mild: 22.2% mod: 52.8% severe: 19.4%			
Jones (2021)	Canada	North America	AAD	January 2019–September 2020	1	131466	PWD: 100%	57.50%	Median: 80				
Kostyál (2021)	Italy Hungary	Europe	CSS	May–July 2020	1	370	Carers: 100%			mild: 0% mod: 44% severe: 42%	88%		spouse: 17% child: 73%
Table 1 Descriptive characteristics of included studies (4/7).													
Kuroda (2022)	Japan	Asia	PR	October 2018–June 2021		1152	PWD: 100%	Before pandemic: 62.3% During pandemic: 58.5%	75+ yrs: BL: 78.0% DL: 76.7%	BL: mild: 56.7% severe: 43.2% DL: mild: 54.3% severe: 45.7%			
Lara (2020)	Spain	Europe	PR			40	PWD: 100%	60%	77.4 (5.3)				
Lion (2022)	Australia	Oceania	QI	July–November 2020		18	Carers: 88.9% PWD: 11.1%	50%	68.5 +‐ 3.5		75%	63.3 +‐ 12.3	31.30%
Llibre‐Rodríguez (2021)	Cuba	North America	PR	BL: 2016–2018 DL: October–November 2020		160	Healthy older adults: 48.8% PWD: 51.3%					BL: 53.6 (56.6) DL: 60.3 (12.2)	BL: spouse: 30.4% DL: spouse: 41.5%
Mackowiak (2021)	Poland	Europe	QI	June–August 2020	1	26	Carers: 80.8% PWD: 19.2%	80%	78+‐6.6		61.90%	63.1 (9.9)	spouse: 28.6% child: 71.4%
Maclagan (2022)	Canada	North America	AAD	March 1 2020– February 21 2021	1, 2	58852	PWD: 100%	56.80%	81.4 (9.7)				
Maggio (2021)	Italy	Europe	CSS	April 1–May 20 2020	1	84	Carers: 100%		62.9 (4.1)		76.20%	45.7 (1.3)	spouse: 27.3% child: 64.3%
Manca (2022)	UK	Europe	CSS	September 2020–March 2021	1	83	Carers: 54.2% PWD: 45.7%	55.50%	70.0 (9.3)		60%	69.2 (10.2)	spouse: 84.4%
Manini (2021)	Italy	Europe	CSS	April 30–June 8 2020		94	Carers: 100%	71.30%	83.2 (5.5)	35.10%	68.10%	64.4 (14.7)	spouse: 44.7% child: 42.6% other family: 7.4% no family: 5.3%
Mohammadian (2022)	Iran	Asia	CSS	September–November 2020	1	40	Carers: 47.5% PWD: 52.5%	42.85%	59.8 (9.7)	mild: 90.5% mod: 9.5% severe: 0	52.60%	56.6 (11.8)	
Table 1 Descriptive characteristics of included studies (5/7).													
Moretti (2021)	Italy	Europe	PR	March 10–July 18 2020		221	PWD: 100%	53.80%	75.6 (6.6)	mild‐mod: 66.1% severe:33.9%			
Morkavuk (2021)	Turkey	Europe	CSS	March 2019–February 2021		202	PWD: 100%	62.80%	BL: 78.5 (7.4) DL: 77.9 (8.2)	BL: mild: 59.3% mod: 35.1% severe: 5.4% DL: mild: 54.1% mod: 35.2% severe: 10.8%			
Oliver (2022)	USA	North America	QI	April 2021		19	Carers: 100%				89.50%	60 (9.5)	spouse: 36.8% child: 47.4%
Paolini (2021)	Italy	Europe	PR	Apr—May 2020	1	38	PWD: 100%	52.60%	81.5 (5.1)				
Penteado (2020)	Brazil	South America	CSS			71	PWD: 100%	69%	76.8 (8.7)	mild‐mod: 35.9% severe: 25%			
Perach (2022)	UK	Europe	PR	July 2019–October 2020	1	175	Carers: 61.7% PWD: 38.2%	58%	79.8 (8.9)		67%	66.1 (13.8)	spouse: 54%
Pickering (2022)	USA	North America	PR	Fall 2019		64	Carers: 100%	44%	78.5 (8.9)		84%	59.6 (13.4)	spouse: 42% child: 48%
Quinn (2022)	UK	Europe	CSS	September 21 2020–April 30 2021	2	242	Carers: 100%	38.80%	75 + yrs: 57.4%		68.20%	75+ yrs: 33.9%	spouse: 86.0%
Rainero (2021)	Italy	Europe	CSS	April 14–April 27 2020		4913	Carers: 100%	59.70%	78.3 (8.2)	mild: 25% mod: 47.8% severe: 27.1%	53.90%	59.3 (13)	spouse: 36% child: 54.5%
Roach (2021)	Canada	North America	QI	April 23–May 21 2020		20	PWD‐carer dyads: 100%	50%	69 (8.3)	mild: 35% mod: 35% severe: 25%			
Rusowicz (2021)	Poland	Europe	CSS	August–October 2020		85	Carers:100%		79 (8.5)		94.1%	51 (11.9)	spouse: 11.8% child: 65.9%
Table 1 Descriptive characteristics of included studies (6/7).													
Russo (2021)	Argentina	South America	CSS	May 2020		119	Carers: 100%	65%	81.2 (7.0)	Mild: 34.5% mod: 31.9% severe: 33.6%	71.90%	58.6 (13.6)	
Sabatini (2022)	UK	Europe	PR	September 2020–April 2021		345	PWD: 100%	48.70%	72.6				
Sánchez‐Teruel (2022)	Spain	Europe	CSS	May 1–May 26 2020	1	310	Carers: 100%				85.80%	46.5 (16.0)	
Sriram (2021)	UK	Europe	QI	October–December 2020		23	Carers:100%				78.30%	range 51‐85	spouse: 47.8% child: 52.1%
Stubbs (2021)	Jamaica	North America	QI	April 2020–May 2021		10	Carers: 100%	80%	75+ yrs: 80%	Mod: 90% severe: 10%	80%	65+ yrs: 20%	spouse: 20% child: 80%
Talbot (2021)	UK	Europe	QI	June–July 2020		19	PWD: 100%	36.80%	62.5 (7.1)				
Theurer (2022)	Germany	Europe	CSS	April–June 2020		165	Carers: 100% (19.7% Carers of PWD)	52.70%	79.4 (8.7)		87.30%	59.8 (9.7)	spouse: 40.7% child: 53.9%
Tondo (2021)	Italy	Europe	PR	July–October 2020	1	132	PWD: 100%	64.40%	79.2 (7.1)				
Tsapanou (2020)	Greece	Europe	CSS	February–June 2020	1	204	Carers: 100%	56.30%	79 (8.9)		75.90%	59 (14)	
Tuijt (2021)	UK	Europe	QI	May–August 2020	1	61	Carers: 50.8% PWD: 49.2%	56.60%			64.50%		spouse: 66.7% child: 71.4% friend: 9.5%
Vaitheswaran (2020)	India	Asia	QI	September 1 2019–February 29 2020	1	31	Carers: 100%	54.80%	70.68 (9.26)	Mild: 51.6% mod: 35.5% severe: 12.9%	51.60%	54.1 (15.0)	spouse: 54.9% child: 41.9%
van Maurik (2020)	Netherlands	Europe	CSS	April 28 2020–July 13 2020		536	PWD: 22% MCI: 9% SCD: 69%	38%	67 (8)				spouse: 92%
Table 1 Descriptive characteristics of included studies (7/7).													
Vislapuu (2021)	Norway	Europe	PR	April–May 2020	1	210	PWD‐carer dyads: 100%	61%	81.8 (6.9)		65.7%	65.5 (12.1)	41.9
Wei (2022)	Germany Netherlands Spain Australia	Europe/Oceania	CSS	April–November 2020		287	Carers:100%	55.40%	73.8 (10.4)		74.90%	57.2 (12.6)	spouse: 44.6% child: 45.6%
Werner (2021)	Israel	Asia	CSS	June–July 2020	1	73	Carers: 100%				86.30%	54.3 (12.3)	spouse: 25.3% child: 74.7%
West (2021)	UK	Europe	QI			15	Carers:73.3% PWD: 26.6%	50%			91%		
Yuan (2021)	China	Asia	CSS	February 11–February 23 2020		787	PWD: 87.3% MCI: 12.7%	65.1%	74.5				
Yuan (2022)	China	Asia	PR	February–October/November 2020		531	PWD: 84.8% Other diseases: 15.3%	65.70%	Median: 74.3 (IQR 9.7)				

**Abbreviations**: AAD, analysis of administrative data; BL, before lockdown/pandemic; CSS, cross sectional survey; DL, during lockdown/pandemic; DLB, dementia with Lewy bodies; MCI, mild cognitive impairment; Mod, moderate; PR, prospective study; PWD, people with dementia; QI, qualitative interview; SCD, subjective cognitive decline; SD, standard deviation.

^a^ Unless indicated otherwise.

### 2.10 Dealing With Missing Data

We contacted study authors to verify key study characteristics, aiming to obtain missing data where possible for inclusion and analysis of the selected study. The remaining missing data were considered in the risk of bias assessment.

### 2.11 Ethical Review

We did not seek ethical approval, as this study did not involve primary data collection.

## 3 Results

**FIGURE 1:**
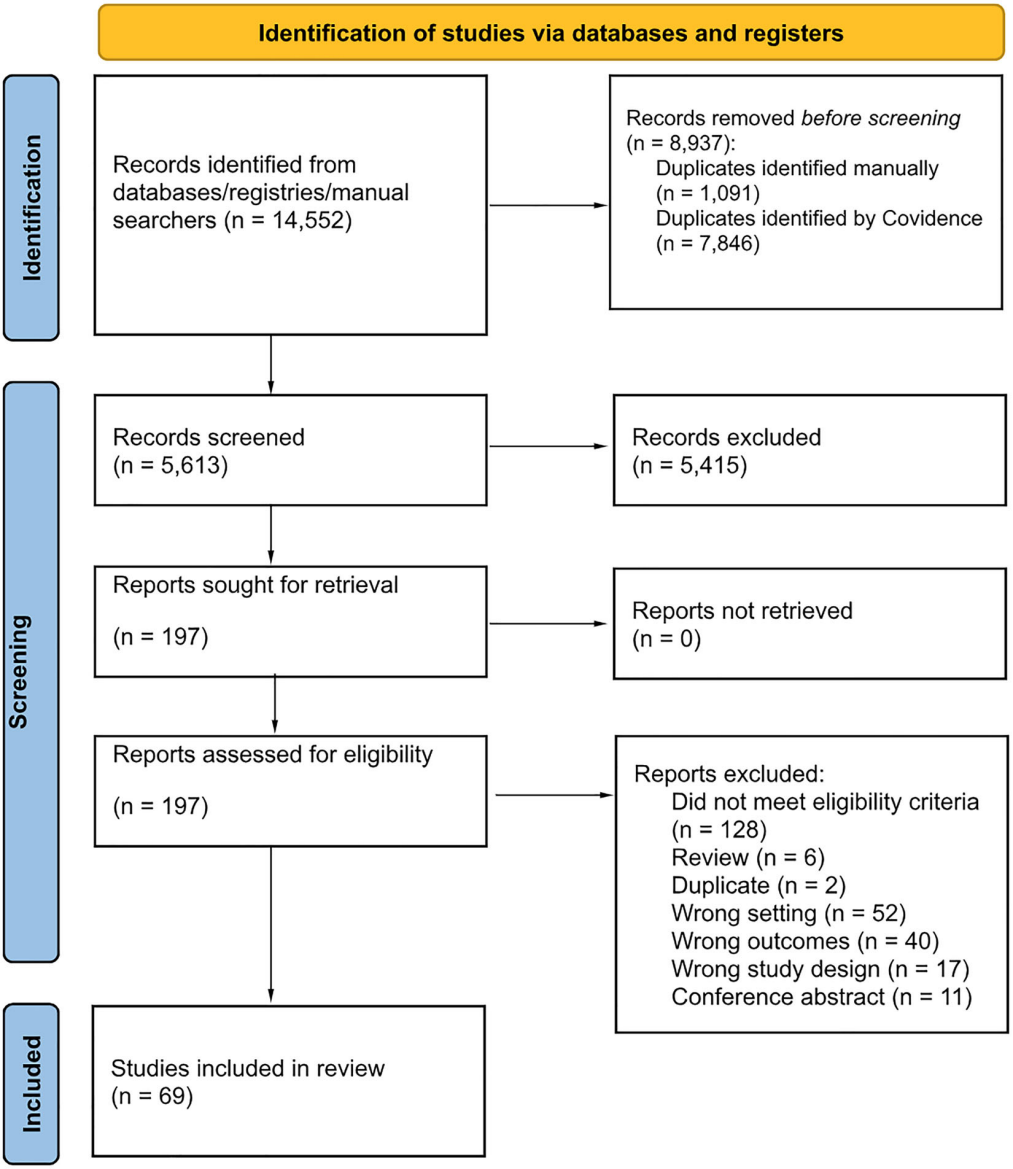
PRISMA flow diagram showing the process of study selection.

A total of 14,552 citations were abstracted from databases searched. After removal of duplicates and screening of titles and abstracts, a total of 197 records underwent full‐text review. Of these, 69 studies met the inclusion criteria and were included in data synthesis (Figure 1).

The vast majority of included studies (74%) were quantitative studies, of which 30 were cross‐sectional studies, 19 were cohort studies, and two were population‐based studies using administrative data. Eighteen of the included studies were qualitative studies. Please see Table 1 for specifics.

### 3.1 Study Characteristics

Table 1 summarizes the study characteristics pertinent to our research questions, including country/region of study origin, sample characteristics, analysis methods, and study results.

### 3.2 Study Origin

Out of a total of 69 studies, 41 were conducted in Europe (France, Germany, Greece, Hungary, Italy, Netherlands, Norway, Poland, Spain, Turkey, and the UK), ten in Asia (China, India, Iran, Israel, Japan, and Kuwait) and North America (Canada, Cuba, Jamaica, and the USA) each, five in South America (Argentina, Brazil, and Chile) and one in Oceania (Australia). Two studies were cross‐country studies and included participants from multiple regions (Europe, Oceania, and North America). No studies were conducted on the African continent (Table 1).

### 3.3 Sample Characteristics

The studies included a total of 209,738 participants. Fifteen studies engaged strictly PWD, 30 studies engaged carers of PWD, and three studies engaged PWD‐carer dyads. The remaining 21 studies included a mix of PWD, MCI, and SCD; carers; and healthy older adults (Table 1).

The studies included a total of 194,532 PWD. The study samples consisted of female participant samples ranging between 37% and 80%, with an average of 56% across studies. Seventeen studies did not explicitly state the sex ratio. The mean age of PWD ranged between 59.8 and 83.2 years of age. The studies included a total of 12,883 carers of PWD. The study samples consisted of female participant samples ranging from 48% to 100%, with an average of 74% across studies. Twenty‐four studies did not explicitly state the sex ratio. The mean age of carers ranged between 45.7 and 70.1 years of age (Table 1).

### 3.4 Risk of Bias and Quality of the Evidence

In this review, the quality of the studies was not part of the inclusion or exclusion criteria, and none of the studies were excluded because of their quality.

**FIGURE 2:**
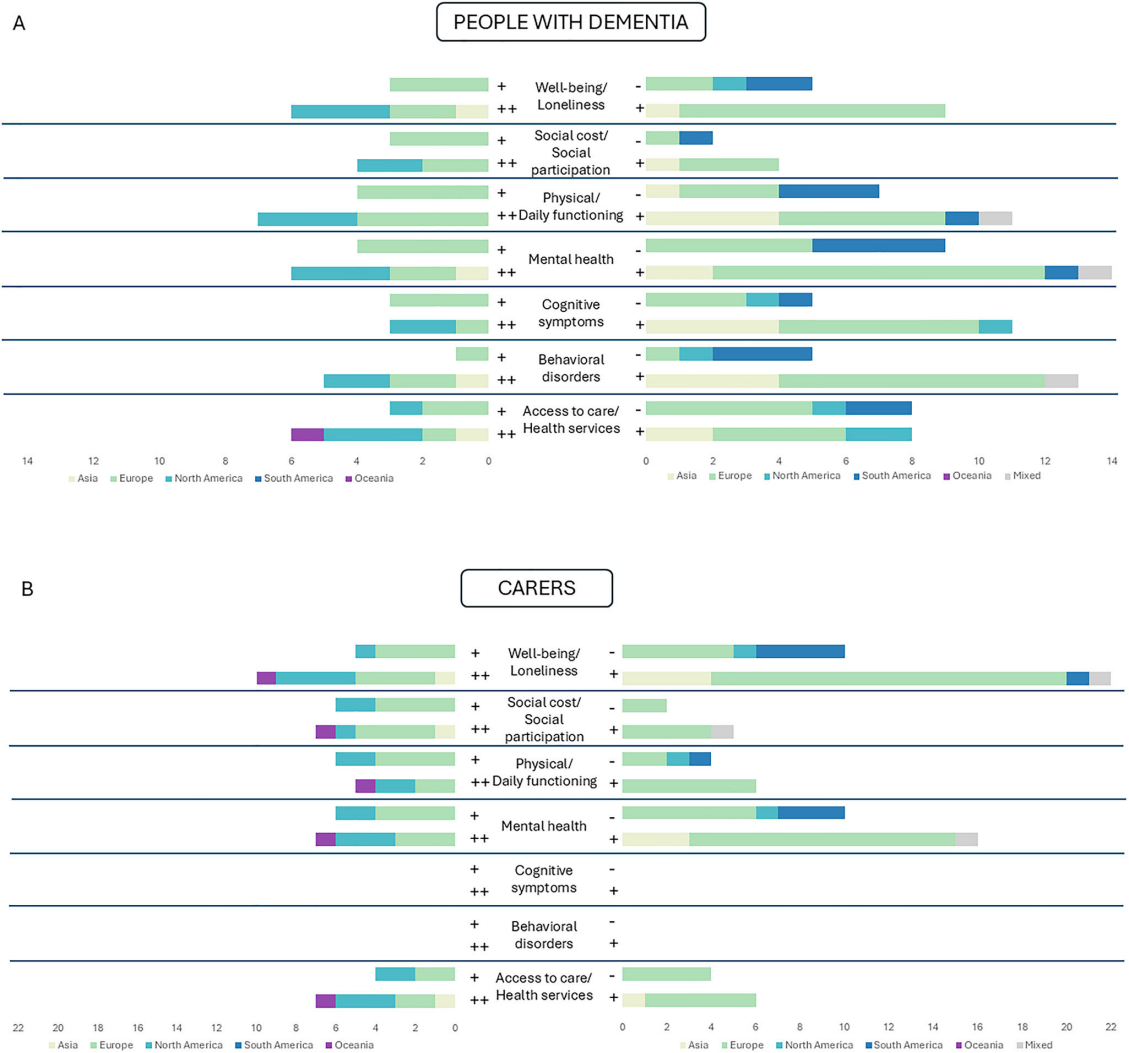
Number of included studies investigating outcomes among people with dementia (Panel 2A) and their carers (Panel 2B). Outcomes were grouped into seven categories: (1) well‐being/loneliness; (2) social cost/social participation; (3) physical/daily functioning; (4) mental health; (5) cognitive symptoms; (6) behavioral disorders; and (7) access to care/health services. Studies were grouped by quality (“−,” low‐quality; “+,” moderate‐quality; “++,”, high‐quality) and study methodology (qualitative, left; quantitative, right). The colors indicate the region where the study was conducted (yellow, Asia; green, Europe; teal, North America; dark blue, South America; purple, Oceania; grey, multiple regions).

We rated the majority of the qualitative studies as high‐quality (“++,” 11 studies) and the remaining studies as moderate‐quality (“+,” 7 studies) (Supplementary Material 2). We rated the majority of the quantitative studies as moderate‐quality (“+”, 36 studies) and the remaining studies as low‐quality (“−”, 15 studies) (Supplementary Material 3). Figure 2A positions a global region comparison based on identified themes for PWD (Figure 2A) and carers (Figure 2B) and quality assessment for qualitative (left) and quantitative (right) studies. None of the outcomes were investigated by all the papers from five regions contributing evidence included in this review (Asia, Europe, North America, South America, and Oceania). There were no qualitative studies originating from South America nor studies including participants coming from multiple regions. There were no quantitative studies originating solely from Oceania (Figure  2).

### 3.5 Physical Health and Daily Routine

In total, 39 studies reported on topics related to physical health and daily routine. These included 15 qualitative studies and 24 quantitative studies (14 cross‐sectional studies, nine cohort studies, and one analysis of administrative data).

#### 3.5.1 Physical Health and Daily Routine of PWD

The qualitative studies reported that the continuing changes in control measures meant ongoing adaptation in everyday activities for PWD (Baumbusch et al. 2022). Inability to go outdoors or meet with others led to changes in diet and (physical) daily activities (Vaitheswaran et al. 2020; Stubbs et al. 2021). Lack of a regular schedule was perceived as distressing and believed to increase symptom progression (Bannon et al. 2021; Talbot and Briggs, 2021). Daily activities such as grocery shopping became more difficult and a source of stress, as people were worried about bringing the virus home (Giebel et al. 2020; Giebel et al. 2021; West et al. 2021). PWD who were able to continue their daily routine seemed to manage well (Tuijt et al. 2021).

The results of qualitative findings were complemented by quantitative studies. Several studies conducted in China, Turkey, Brazil, Argentina, the UK, and Italy reported a decline in daily functioning, either measured as physical activity, functional dependency/limitations, personal care, or mobility (Barguilla et al. 2020; Chen et al. 2021; Helvaci Yilmaz et al. 2021; Penteado et al. 2020; Manca et al., 2021; Tsapanou et al. 2021; Cohen et al. 2020b; Azevedo et al. 2021; Maggio et al. 2021). One Chinese study looked at the prevalence of malnutrition and found that only 3.3% of 787 PWD were malnourished, but half were at risk of being malnourished (Yuan et al. 2021). Studies were also consistent in that 37%–61% of carers reported a decline in the health of the PWD (Kostyál et al. 2022; Rusowicz et al. 2021).

Sleep problems were not mentioned in the qualitative studies but were reported in quantitative studies. Studies in Asia, South America, Europe, and Oceania all found that the prevalence of sleep problems was higher during or after lockdown than before (Azevedo et al. 2021; Gan et al. 2021; Yuan et al. 2022; Wei et al. 2022).

#### 3.5.2 Physical Health and Daily Routine of Carers

In the qualitative studies, carers reported having to adapt their own lives (e.g., quit jobs or work remotely) to take care of the PWD (Stubbs et al. 2021; Bannon et al. 2021; Giebel et al. 2021). Reduced work hours led to financial worries (Stubbs et al. 2021; Bannon et al. 2021; Giebel et al. 2021). A new role for carers was to ensure the PWD was adhering to the control measures (Baumbusch et al. 2022; Stubbs et al. 2021), which was often perceived as challenging (Baumbusch et al. 2022; Lion et al. 2022). With the increased care duties and reduced formal home care services, the carers reported stress around coping with day‐to‐day activities (Roach et al. 2021), even limiting their ability to look after themselves (e.g., stopping exercise) (Lion et al. 2022). Through its impact on the PWD, lack of a regular schedule also affected the health of the carers (e.g., chest pain) (Baumbusch et al. 2022; Maćkowiak et al. 2021; Sriram et al. 2021).

Quantitative studies confirmed the findings of qualitative studies in that the lockdown had a substantial impact on the daily lives of care partners (Rusowicz et al. 2021; Theurer et al. 2022; Rainero et al. 2021). The time spent caring increased for most carers (Azevedo et al. 2021, Kostyál et al. 2022) and there were proportionally more carers spending >10 h caring per day (Gamble et al. 2022). The caregiving required more physical effort, and carers felt more tired (Azevedo et al. 2021), consequently increasing the burden of care (Barguilla et al. 2020; Tsapanou et al. 2021; Cohen et al. 2020b; Yuan et al. 2022; Theurer et al. 2022; Russo et al. 2021; Cohen et al. 2020b). Nearly a third of 4913 Italian carers and 12% of 165 German carers reported a reduction in time available for their own activities (Theurer et al. 2022; Rainero et al. 2021).

### 3.6 Cognitive Functioning

In total, 28 studies reported on topics related to cognitive functioning. These included 9 qualitative studies and 19 quantitative studies (12 cross‐sectional studies and 7 prospective cohort studies).

#### 3.6.1 Cognitive Functioning of PWD

In qualitative interviews, care partners expressed that they felt the cognitive functioning of PWD declined more rapidly during the lockdown than before (Talbot and Briggs, 2021; Tuijt et al. 2021; Giebel et al. 2021; Lion et al. 2022; Roach et al. 2021; Geyer et al. 2020). Most studies attributed this accelerated decline to a reduction in cognitive, mental, or social activation (Talbot and Briggs, 2021; Roach et al. 2021; Geyer et al. 2020), confusion due to not understanding the control measures (Bannon et al. 2021; Giebel et al. 2021; Cipolletta et al. 2023), and lack of routine (Giebel et al. 2021). In contrast, in one study, carers argued that the accelerated decline could be explained by the progressive nature of dementia and/or their increased proximity to the PWD, such that they noticed the decline more (Tuijt et al. 2021).

Findings from quantitative studies used a variety of study designs to examine the (rate of) decline in cognition during lockdown. Most studies were cross‐sectional surveys asking PWD or carers about subjective (decline in) cognitive functioning of the PWD (Barguilla et al. 2020; Helvaci Yilmaz et al. 2021; Penteado et al. 2020; Manca et al.; Tsapanou et al. 2021; Azevedo et al. 2021; Rainero et al. 2021; van Maurik et al. 2020). They reported subjective worsening of cognition in 35%–73% of PWD. Two studies that measured cognitive functioning before and during lockdown found a similar magnitude of decline in Mini Mental State Examination (MMSE) scores, with a mean decline of 1.61 ± 3.4 in 105 Chinese people with Alzheimer's disease (AD) (Chen et al. 2021) and 1.99 ± 0.42 in 38 Italian people with AD (Paolini et al. 2021). Only two studies measured the *rate* of decline. In 36 PWD from Kuwait, the mean monthly rate of decline was 0.53 ± 0.3 points during lockdown, which was significantly higher than before lockdown (0.2 ± 0.1, *p* < 0.001) (Ismail et al. 2021). In 40 PWD from Italy, the within‐group annual rate of decline was greater from 2019 to 2020 than from 2017 to 2019 (*p* < 0.05) (Tondo et al. 2021). In summary, despite differences in study design, findings consistently point toward a decline in cognitive functioning during lockdown. There is sparse evidence suggesting that the rate of decline was more rapid during than before lockdown.

### 3.7 Behavioral Problems, Mental Health, and Wellbeing

In total, 66 studies reported on topics related to behavioral problems, mental health, and well‐being. These included 20 qualitative studies and 46 quantitative studies (28 cross‐sectional studies and 18 prospective studies).

#### 3.7.1 Behavioural Problems, Mental Health, and Wellbeing of PWD

In qualitative interviews, both PWD and carers indicated that the control measures negatively affected the mental health and well‐being of PWD and led to increased behavioral problems (Vaitheswaran et al. 2020; Talbot and Briggs, 2021; Geyer et al. 2020; Cipolletta et al. 2023; Oliver et al. 2022). Behavioral problems included increased apathy, anxiety, restlessness, irritability, sleep disturbances, aggression, and agitation (Vaitheswaran et al. 2020; Bannon et al. 2021; Giebel et al. 2021; West et al. 2021; Tuijt et al. 2021; Geyer et al. 2020). This was mainly attributed to reduced social contacts, closure of day care services, others wearing masks causing confusion, and worries about their loved one's health and well‐being (Vaitheswaran et al. 2020; Bannon et al. 2021; Talbot and Briggs, 2021; Giebel et al. 2021; West et al. 2021; Giebel et al. 2021; Geyer et al. 2020). Social isolation and lack of meaningful activities were often cited as the main causes for increased feelings of loneliness (Talbot and Briggs, 2021; Maćkowiak et al. 2021; Geyer et al. 2020), and the inability to understand the need for control measures further exacerbated these problems (Giebel et al. 2021; West et al. 2021; Geyer et al. 2020). The impact on mental health was even stronger in PWD who were aware of their declining independence (Tuijt et al. 2021). Coping strategies such as using alternate communication methods to maintain social contacts (e.g., video calls) were often not available or feasible (Geyer et al. 2020).

In contrast, some studies reported positive consequences of the pandemic, including time to return to past hobbies, learn new skills, and a welcome break from the busy and therefore stressful normal routine (Talbot and Briggs, 2021; West et al. 2021). Practical support from family in adapting to the situation provided a sense of security and helped maintain well‐being (Stubbs et al. 2021; Tuijt et al. 2021).

Quantitative studies showed higher prevalence of neuropsychiatric symptoms during the pandemic than before the pandemic (Chen et al. 2021; Manca et al., 2021; Gan et al. 2021; Rainero et al. 2021; van Maurik et al. 2020; Kuroda et al. 2022; Moretti et al. 2021; Boutoleau‐Bretonnière et al. 2020; Lara et al. 2020; Manini et al. 2021) and lower prevalence after lifting of quarantine (Yuan et al. 2022; Moretti et al. 2021). Studies that compared prevalence of symptoms later on in the pandemic with pre‐ or early stages of the pandemic found a decrease in prevalence as the pandemic continued (Bakker et al. 2022; Sabatini et al. 2022), although changes were not always statistically significant (Giebel et al. 2021). In the early stages of the pandemic, 44%–65% of PWD reported increases in symptoms, which was fairly consistent across countries given variations in study design (Barguilla et al. 2020; Chen et al. 2021; Manca et al., 2021; Rainero et al. 2021; Cohen et al. 2020a; van Maurik et al. 2020; Kostyál et al. 2021). The most commonly reported symptoms included anxiety, depression, apathy, agitation, irritability, and sleep problems/nighttime behavior (Barguilla et al. 2020; Bakker et al. 2022; Chen et al. 2021; Helvaci Yilmaz et al. 2021; Penteado et al. 2020; Manca et al., 2021; Cohen et al. 2020a; Azevedo et al. 2021; Yuan et al. 2021; Yuan et al. 2022; Wei et al. 2022; Russo et al. 2021; van Maurik et al. 2020; Kuroda et al. 2022; Lara et al. 2020; Manini et al. 2021; Giebel et al. 2021).

PWD seemed to experience less worry or stress related to the COVID‐19 outbreak itself than healthy controls (Hashimoto et al. 2020). In 787 Chinese PWD, only 8.6% worried about the outbreak. However, among those who did worry, the prevalence of anxiety and nervousness was high (83.8%) (Yuan et al. 2021). In a sample of 38 Italian PWD, stress levels increased from the start of the lockdown to 2 weeks later, then stabilized another 2 weeks later. (p<0.05) (Paolini et al. 2021).

During the lockdown, social contact decreased relative to before the lockdown (Chen et al. 2021; van Maurik et al. 2020). Studies that reported on loneliness all showed increases in loneliness during lockdown relative to before lockdown, but with prevalences varying from 17% in the Netherlands to 31% in South America and 95% in Iran (Bakker et al. 2022; Azevedo et al. 2021; Grycuk et al. 2022).

Two UK studies and one Spanish study found no significant changes in quality of life from pre‐lockdown to during lockdown (measured as self‐reported and/or proxy‐reported Dementia Quality of Life (DEMQOL) or EuroQol Five‐Dimensions (EQ‐5D)) (Lara et al. 2020; Hicks et al. 2022; Daley et al. 2022). In contrast, another UK study found that mental wellbeing (measured with the Short Warwick‐Edinburgh Mental Wellbeing Scale (SWEMWBS)) gradually improved as the lockdown progressed (Giebel et al. 2021), and an Italian study found that the quality of life (measured with the Quality of Life in Late‐stage Dementia (QUALID) scale) first declined during lockdown and then recovered after lockdown to pre‐lockdown levels (Moretti et al. 2021).

#### 3.7.2 Behavioral Problems, Mental Health, and Wellbeing of Carers

Stress and concerns were common themes across qualitative studies, for which a number of causes and consequences were cited. Care partners were concerned about themselves or the PWD being infected with COVID‐19 and being isolated, hospitalized, or even losing their life, and being unable to provide the required care (Vaitheswaran et al. 2020; Stubbs et al. 2021; Bannon et al. 2021; West et al. 2021; Lion et al. 2022; Roach et al. 2021; Maćkowiak et al. 2021; Geyer et al. 2020; Cipolletta et al. 2023). These concerns were exacerbated by lack of information from governments or health services (Giebel et al. 2021; Lion et al. 2022). Carers experienced an increased care burden as support from family or professional care dropped away (Lion et al. 2022; Roach et al. 2021; Geyer et al. 2020; Cipolletta et al. 2023; Flemons et al. 2022). Carers experienced extra stress if they (a) had to take over tasks previously done by care professionals that they did not feel qualified for (Giebel et al. 2020), (b) felt forced to move in with the PWD (Giebel et al. 2020), or (c) feared or experienced unemployment or economic difficulties (Roach et al. 2021; Cipolletta et al. 2023). Adhering to the control measures, (repeatedly) explaining these to the PWD, and adjusting to the routine of the PWD added to the care burden and subsequently caused stress and fatigue (Baumbusch et al. 2022; Giebel et al. 2021; West et al. 2021; Tuijt et al. 2021; Maćkowiak et al. 2021; Cipolletta et al. 2023). The pandemic situation also confronted carers with the need for care‐planning conversations with the PWD, family, and medical team. Making such decisions in a time of heightened risk and reduced availability of services exacerbated their stress (West et al. 2021). Other sources of stress were the lack of social interactions and not having an outlet or time for hobbies (Baumbusch et al. 2022; Giebel et al. 2020; West et al. 2021; Geyer et al. 2020; Cipolletta et al. 2023; Oliver et al. 2022; Flemons et al. 2022).

For some carers, the stress led to panic attacks, anxiety, depression, fatigue; or feelings of loneliness (Baumbusch et al. 2022; Giebel et al. 2020; West et al. 2021; Geyer et al. 2020; Oliver et al. 2022). The stress also negatively affected the care relationship, triggering behavioral problems in the PWD (Bannon et al. 2021; Geyer et al. 2020; Cipolletta et al. 2023). This caused a vicious cycle, with more stressed carers leading to more behavioral problems in the PWD leading to more stressed carers, etcetera (Giebel et al. 2021). In contrast, two studies reported that spending more time with the PWD helped strengthen the care relationship (Bannon et al. 2021; Sriram et al. 2021).

Some studies discussed coping strategies. Spirituality, meaningful activities (e.g., gardening, exercise), enjoying good weather, and self‐care provided relief and a sense of purpose (Stubbs et al. 2021; Geyer et al. 2020). Maintaining routine/daily structure, creatively adapting to the situation, and hiring paid care helped to cope (Stubbs et al. 2021; Lion et al. 2022; Maćkowiak et al. 2021; Geyer et al. 2020). Carers tried to assist the PWD with alternate forms of communication, mainly digital tools, to compensate for the lack of in‐person social connections, but this was not satisfactory to the same degree (West et al. 2021; Tuijt et al. 2021; Geyer et al. 2020; Cipolletta et al. 2023) and also not accessible to all (Stubbs et al. 2021; Flemons et al. 2022).

The quantitative studies confirmed the findings from qualitative studies in that the control measures negatively affected the care partners’ mental health. Studies reported increases in depressive symptoms, anxiety, irritability, and stress (Manca et al., 2021; Azevedo et al. 2021; Maggio et al. 2021; Wei et al. 2022; Theurer et al. 2022; Rainero et al. 2021; Manini et al. 2021; Kostyál et al. 2021; Altieri and Santangelo, 2021; Bussè et al. 2022), as well as concerns related to the pandemic, the health of the PWD, and their own health (Cohen et al. 2020b; Rusowicz et al. 2021; Theurer et al. 2022). During the pandemic, the mean scores for depression, but not anxiety, were significantly higher in carers than in non‐carers (Bussè et al. 2022). Factors associated with mental health problems in carers included disease progression, neuropsychiatric symptoms and health of the PWD, perceived care burden, informal support network, paid home care, day care, carer‐PWD relationship, pandemic‐related concerns, social isolation, and hobbies (Bakker et al. 2022; Maggio et al. 2021; Yuan et al. 2021; Rusowicz et al. 2021; Wei et al. 2022; van Maurik et al. 2020; Boutoleau‐Bretonnière et al. 2020; Manini et al. 2021; Kostyál et al. 2021; Bussè et al. 2022; Alexopoulos et al. 2021; Llibre‐Rodriguez et al. 2021).

Several studies reported either an increase in levels of loneliness or high levels of loneliness during the pandemic (Azevedo et al. 2021; Theurer et al. 2022; Mohammadian et al. 2022; Grycuk et al. 2022). Factors associated with higher levels of loneliness included anxiety, having more formal activities during the day, relationship to the PWD, co‐residing with the PWD, health of the PWD, cognitive functioning of the PWD, and caregiving burden (Grycuk et al. 2022; Perach et al. 2022). At the same time, loneliness contributed to increased perceived caregiving burden (Grycuk et al. 2022).

A series of UK studies found that approximately half of carers reported high levels of well‐being (Gamble et al. 2022; Quinn et al. 2022), with no or modest decline in quality of life (measured with the Dementia Quality of Life of Carers (DEMQOL‐C) and self‐rated quality of life) during the pandemic (Hicks et al. 2022; Daley et al. 2022; Quinn et al. 2022). In contrast, a survey among 84 Italian carers found that, on average, participants scored very low on physical and mental well‐being (measured with the 12‐item Short Form Survey SF‐12) (Maggio et al. 2021). Factors associated with well‐being included resilience, self‐efficacy, coping strategies, employment status, type of dementia, dementia severity, and type of dwelling (Daley et al. 2022; Sánchez‐Teruel et al. 2022).

As stated earlier, many studies reported an increase in caregiver burden, which plays a key role in the mental health and well‐being of carers. The negative impact of the care burden may be mitigated by coping strategies. Studies showed that carers indicated needing a break (34.5% of a Chinese sample), more support (29.1% in a Dutch sample), or more time for themselves (50% of a Turkish sample) (Yuan et al. 2021; Gamble et al. 2022; van Maurik et al. 2020). Of note, an Italian study concluded that participants mostly used maladaptive coping strategies, such as avoidance strategies, but these strategies did not affect the stress level of caregivers (Maggio et al. 2021).

### 3.8 Social and Economic Consequences

In total, 28 studies reported on topics related to social and economic consequences of the control measures. These included 17 qualitative studies and 11 quantitative studies (seven cross‐sectional and four cohort studies).

Findings from qualitative and quantitative studies consistently indicated that decreased social life and support was a key issue for both PWD and their carers (Vaitheswaran et al. 2020; Stubbs et al. 2021; Bannon et al. 2021; Giebel et al. 2021; Wei et al. 2022; Cohen et al. 2020b; Cipolletta et al. 2023). As described above, this had a negative impact on their mental health and well‐being. For care partners, lack of informal support meant that they had to take on more caregiving tasks, particularly if paid care had also dropped away.

The pandemic had financial consequences for many people, but there were some consequences that were specific for PWD and their carers, namely: (1) loss of income due to the need to reduce work hours or even quit their job to care for the PWD (Stubbs et al. 2021; Rusowicz et al. 2021; Cipolletta et al. 2023). Carers, particularly those whose job situation did not allow for remote work, had to choose between care duties and income (Stubbs et al. 2021). And (2) increased costs due to having to hire paid care (Stubbs et al. 2021; Cipolletta et al. 2023) and purchasing hygiene products to reduce the risk of infection (e.g., masks, sanitizer) (Baumbusch et al. 2022) or other products they were already using (e.g., wipes, medication) that went up in price due to scarcity (Baumbusch et al. 2022; Vaitheswaran et al. 2020). These financial consequences particularly affected people with low incomes, therefore increasing already existing inequalities (Stubbs et al. 2021; Giebel et al. 2021).

### 3.9 Access to Health Services

In total, 39 studies reported on topics related to social and economic consequences of the control measures. These included 16 qualitative studies and 23 quantitative studies (17 cross‐sectional studies, 4 prospective cohort studies, and 2 studies analyzing administrative data).

Qualitative studies reported that PWD and their carers were greatly affected by the reduced formal health and social care services, such as closure of day care centres, closure of respite care, cessation of physio‐ and occupational therapy, and decrease in domestic and/or home care and peer support groups (Baumbusch et al. 2022; Bannon et al. 2021; Giebel et al. 2020; West et al. 2021; Lion et al. 2022; Maćkowiak et al. 2021; Geyer et al. 2020; Cipolletta et al. 2023). As the entire health care system was focused on controlling the pandemic, even access to basic health care services was limited. For example, it was difficult to get doctors’ appointments or access to medication (Vaitheswaran et al. 2020; Stubbs et al. 2021; West et al. 2021; Maćkowiak et al. 2021; Sriram et al. 2021; Cipolletta et al. 2023; Morkavuk et al. 2021). Offered solutions such as telemedicine were not always accessible or feasible for PWD (Bannon et al. 2021; Giebel et al. 2021; Lion et al. 2022; Roach et al. 2021; Maćkowiak et al. 2021; Sriram et al. 2021; Oliver et al. 2022). As already alluded to above, this led to (1) disruption of routine and less activation of the PWD, possibly leading to accelerated disease progression, and (2) increased care burden, with no prospect of a break in care duties.

When home or day care services were available, care partners sometimes chose to stop the care due to difficulties with ensuring care professionals were adhering to the control measures or due to fear of bringing the virus home (Giebel et al. 2020; West et al. 2021; Maćkowiak et al. 2021; Cipolletta et al. 2023). A few studies also noted that lack of information on available support was an issue (Lion et al. 2022; Flemons et al. 2022).

Quantitative studies confirmed the findings in qualitative studies, suggesting care use was lower during the pandemic and therapies stopped (Cohen et al. 2020b; Theurer et al. 2022; Russo et al. 2021; Cohen et al. 2020b; Kuroda et al. 2022; Giebel et al. 2021; Vislapuu et al. 2021; Jones et al. 2021). The only exception is a USA study of 64 PWD, which reported no significant difference in the number of days receiving formal care during versus pre‐pandemic (Pickering et al. 2022). However, these differences are likely explained by variations in how the questions were asked. In time‐series analyses of administrative care use data in Canada, the home care visits dropped by 16% at the start of the pandemic (mid‐March 2020) but recovered to normal levels by September 2020 (Jones et al. 2021). Similarly, use of therapies dropped by 50% at the start of the pandemic and exceeded pre‐pandemic levels by September 2020 (Jones et al. 2021). These findings were partly confirmed in a second Canadian study using administrative health services data, which also found a drop in visits per 100 PWD (2.41 in the reference period versus 1.20 in the pandemic period), but in that study, the number of visits did not return to pre‐pandemic levels after the second wave (Maclagan et al. 2022).

Two quantitative studies reported contrasting socioeconomic differences in access to care. In 73 Israeli carers, those with higher levels of education or higher income were less likely to report forgone care (Werner et al. 2021), while in a time‐series analysis of administrative data of 131,466 Canadian PWD, the decline in personal care was greater in those living in more affluent areas (Jones et al. 2021). Hence, the potential modifying effect of socio‐economic factors on the impact of the control measures on health services use remains unclear or could potentially reflect differences across countries.

### 3.10 Country‐level Differences

#### 3.10.1 Physical Health and Daily Routine

While studies conducted in different countries measured different outcomes, overall, the tendency of decline in physical health and changes in daily routine and increased care needs were consistent across countries and regions. No notable differences between countries were found across the studies, apart from some reflections in qualitative studies on services that were specific to a given country (e.g., food packages handed out in the UK).

#### 3.10.2 Cognitive Functioning

The qualitative studies were all conducted in Europe and North America, with consistent findings across these two regions. Quantitative studies were conducted in all regions except Oceania and Africa. We observed consistency in findings across study designs and countries.

#### 3.10.3 Behavioral Problems, Mental Health, and Wellbeing

While individual studies from different countries highlighted different elements, the collection of findings tells a coherent story that illustrates a uniform impact of the control measures on the mental health and well‐being of both the PWD and their carers. These findings seem consistent across countries and regions. The long‐term impact of the control measures on the mental health of the care partners remains unclear.

#### 3.10.4 Social and Economic Consequences

While no notable regional differences could be observed, financial consequences came up more frequently in studies conducted in low‐ and middle‐income countries such as India and Jamaica. A few studies pointed toward differences in people in lower or higher socioeconomic positions. The financial consequences were felt more by people with low incomes, while people in higher socio‐economic positions had more access to social and other resources to cope with the situation (Stubbs et al. 2021; Giebel et al. 2021).

#### 3.10.5 Access to Health Services

While there is consistent evidence that the control measures had a significant impact on access to health and care services for PWD in all countries (except the US), the magnitude of this impact is likely to differ across countries given the differences in availability of health services for PWD, as well as cultural differences in seeking formal support. However, direct or indirect country comparisons are hampered by the variation in study designs and measurement of service use.

## 4 Discussion

### 4.1 Principal Findings

Pre‐pandemic studies have reported that PWD often experience social isolation and loneliness, physical and mental adversities, challenges with relationships and intimacy, and reduced quality of life. (Stubbs et al. 2021; Bannon et al. 2021) Therefore, we anticipated exacerbation of adverse outcomes related to the control measures during the COVID‐19 pandemic. Adverse outcomes were consistently reported in the scientific evidence included in this review, spanning many countries and regions. The impact of the pandemic on carers of PWD was also largely uniform, who often reported living with anxiety and fear and experiencing difficulties balancing caregiving challenges with their own needs.

The experiences of PWD and carers that emerged in the qualitative research were of a diverse and personal nature. Some PWD were able to continue regular daily activities at home, such as gardening, doing crosswords, or reading, and seemed to manage the control measures well, especially when they were not excessively complex. Some carers and PWD reported that their diets had improved or become healthier during the pandemic as they were able to make fresh meals. On the other hand, carers supporting a person with FTD rather than with AD reported greater burden in the included studies, likely because people with FTD are known to experience more behavioral and psychological symptoms (Rainero et al. 2021) and reduced access to formal care support (Boutoleau‐Bretonnière et al. 2020). In some cases, there was a change in the person delivering the care, from a spouse to an adult child (Paolini et al. 2021), or a lack of family support to the main carer (Cohen et al. 2020b). Synthesis of qualitative research raises important discussions about variability in the impact of control measures and the role of a person's living environment and social influences (i.e., PROGRESS‐Plus parameters) (Sant'Ana et al. 2024) in their experiences.

The majority of the studies included in this review came from high income countries. Among included studies, financial consequences came up more frequently in studies conducted in low‐ and middle‐income countries, such as India and Jamaica (Vaitheswaran et al. 2020; Stubbs et al. 2021). A few studies pointed toward differences in people in lower or higher socio‐economic positions (Baumbusch et al. 2022; Jones et al. 2021). It is probable that those with lower income were more affected due to increased likelihood of holding jobs that put them in a vulnerable position (unable to work from home; higher risk of losing job; front‐line jobs with higher risk of infection), indicating that social capital and socioeconomic status play an important role on brain health. Furthermore, regarding the influence of race and ethnicity, the research conducted by West et al. included black, Asian, and minority ethnic groups in the United Kingdom (West et al. 2021). Findings were similar to those in other studies, suggesting little ethnic or cultural differences, or a failure of studies to capture this in their analyses. Research sensitive to the person's living environment and social capital can enhance our understanding of the needs of PWD and their carers within and between countries, and has the potential to foster complex discussions with research, policy, and practice implications. This is particularly relevant for countries in Africa, since no studies included in this review originated from this region.

### 4.2 Strengths and Limitations

Our review has several strengths. First, we did not limit our searches to English language studies only; rather, we included studies published in multiple languages. This approach allowed for extraction of data collected in non‐English‐speaking countries with limited resources to disseminate their work with the larger scientific community. Second, we included both quantitative and qualitative research. Our approach took into account the advantages of the two types of research. We collected topics from open‐ended questions and qualitative research to better understand the lived experience and the societal impacts of the pandemic, while also synthesizing results from quantitative studies, which used standardized measures to study multiple associations relevant to our research objectives. To the best of our knowledge, this is the first comprehensive systematic review on this topic aggregating data from both qualitative and quantitative studies. This is also the first systematic review that reviewed evidence through a global lens. Finally, our multiple subgroup analyses with a focus on relevant endpoint and visual data presentation allowed for nuanced comparisons across studies, countries, and global regions.

We acknowledge several limitations. First, the heterogeneity in the included studies prevented us from conducting a formal assessment of publication bias. Heterogeneity was observed at multiple levels (i.e., sample characteristics, clinical settings, spectrum of studied comorbidities, reporting, statistical approaches, and outcome measurements); even within the same country and group of people the strength of association varied. Second, prior to data extraction, we decided on the confounding variables that were considered as important to studied outcomes. However, many of these variables were missing. The type of dementia and severity were not always reported in included studies, despite being important variables that could influence the outcomes for PWD and their carers. A concern inherent to all dementia research is that cognitive impairments of PWD may affect their ability to understand survey questions, and carers who provide care to PWD may have influenced responses of PWD in studies involving dyads, consequently impacting the data collected. Access to and proficiency with technology required to complete self‐report surveys during the pandemic may have led to biased samples and limited generalizability to people who are living with more advanced stages of dementia, those facing communication barriers, and those with limited access to or proficiency with technology (Zhang et al. 2023).

The quantitative studies included in this systematic review reported associations while considering various confounding parameters. It is possible that the associations between confounding parameters and the outcomes of interest can be attenuated due to “over‐controlled” variables (Table 1). For instance, since there is a possible causal relationship between age, gender, insomnia, dementia, and studied outcomes, including them in a model might have attenuated the association between them and the studied outcomes because they might be in the causal pathway to many adverse effects (Henry et al. 2019; Prather et al. 2015). However, we observed consistency in findings, which strengthens the robustness of results and mitigates this concern to some extent. Finally, we included studies up to July 2022; thus, the results are limited to the earlier stages of the pandemic (wave 1 and 2). The perspectives of PWD and carers may have shifted over time as the pandemic progressed and control measures changed. However, the control measures were most strict in the earlier stages of the pandemic, suggesting that our review captured the major consequences of such measures.

### 4.3 Implications of the Results for Practice, Policy, and Future Research

Results from our systematic review have highlighted challenges with access to health services, (exacerbated) deterioration of physical, cognitive, and mental function, as well as social and financial adversities faced by PWD and their carers living at home during the COVID‐19 pandemic. It is essential that, globally, decision‐makers understand the needs of PWD when implementing control measures. People with dementia and their carers represent a heterogeneous group of people across countries and communities; despite that, we did not identify many contradictory results in the included literature. Social equity parameters should be considered and implemented in future studies to identify variables within populations of interest that could be associated with a poorer overall experience during the pandemic and lead to studies of higher quality to enhance certainty in the results.

The impact of COVID‐19 should be assessed separately according to various living environments to identify more at‐risk groups and regions, particularly those without evidence included in this review (i.e., Africa). Future studies should also analyze different protective and risk factors among PWD and their carers. For example, it would be important to compare the effect of living alone versus living with a carer, of being younger in age versus older, or of having greater community support versus less. Moreover, future studies can delve deeper into resilience, regulation of emotions, and coping strategies of PWD and their carers. Micro‐, meso‐, and macro‐level policies should be established to ensure that PWD and their carers are trained on how to use communication technologies to enable them to preserve social links, uphold family bonds, and maintain the ability to give or receive needed care when faced with isolation and protective measures during future pandemics or crises.

## 5 Conclusions

The results of this systematic review highlight the impact of public health measures on the vulnerability of PWD living at home and their carers across 27 countries and five regions globally and suggest the need for proactive planning of prevention measures to mitigate risks in anticipation of potential public health threats. Future studies should evaluate the long‐term effect of control measures, particularly during the subsequent waves of the pandemic, on PWD living at home and their carers.

## Author Contributions

**Yaohua Chen**: methodology, formal analysis, investigation, critical appraisal, validation, scientific messages, writing – original draft, review and editing, funding acquisition. **Tatyana Mollayeva**: methodology, formal analysis, investigation, critical appraisal, validation, scientific messages, writing – original draft, review and editing, funding acquisition. **Rachael Fitzpatrick**: methodology, formal analysis, investigation, critical appraisal, validation, writing – original draft, review and editing. **Thaisa Tylinski Sant'Ana**: investigation, data visualization, supplementary material, writing – review and editing. **Francesca R. Farina**: methodology, formal analysis, investigation, critical appraisal, validation, scientific messages, writing – review and editing, funding acquisition. **D. Swiatek**: methodology, formal analysis, investigation, critical appraisal. **K. Sopidou**: methodology, formal analysis, investigation, critical appraisal. **E. Tabilo**: methodology, formal analysis, investigation, critical appraisal. **M. Betka**: methodology, formal analysis, investigation, critical appraisal. **Iracema Leroi**: methodology, critical appraisal, validation, scientific messages, review and editing. **T. Leon**: formal analysis, writing – original draft, review and editing. **Geeske Peeters**: methodology, formal analysis, investigation, critical appraisal, validation, scientific messages, writing – original draft, review and editing, funding acquisition. All authors contributed to the article, as well as read and approved the final manuscript.

## Funding

This research was supported by the team grant: JPND Call for Expert Working Groups: The Impact of COVID‐19 on Neurodegenerative Diseases in partnership with the CIHR‐Institute of Aging; Public Health Agency (CIHR #02342‐000). TM was supported by the Canada Research Chairs Program (Neurological Disorders and Brain Health, CRC‐2021‐00074). This work emerged through liaising with global stakeholders at the GBHI. The funders had no role in the study's design, data collection and analysis, decision to publish, or preparation of the manuscript.

## Conflicts of Interest

The authors declare no conflicts of interest.

## Supporting information




**Supplementary Materials**: brb371100‐sup‐0001‐SuppMat.pdf

**Supplementary Materials**: brb371100‐sup‐0002‐SuppMat.docx

**Supplementary Materials**: brb371100‐sup‐0003‐SuppMat.docx

## Data Availability

The data that support the findings of this study are available from the corresponding author upon reasonable request.

## References

[brb371100-bib-0001] Alexopoulos, P. , R. Soldatos , E. Kontogianni , et al. 2021. “COVID‐19 Crisis Effects on Caregiver Distress in Neurocognitive Disorder.” Journal of Alzheimer's Disease 79, no. 1: 459–466. 10.3233/JAD-200991. 33185608

[brb371100-bib-0002] Alkhaldi, G. , G. S. Aljuraiban , S. Alhurishi , et al. 2021. “Perceptions Towards COVID‐19 and Adoption of Preventive Measures Among the Public in Saudi Arabia: A Cross Sectional Study.” BMC Public Health [Electronic Resource] 21, no. 1: 1251. 10.1186/S12889-021-11223-8. 34187425 PMC8240080

[brb371100-bib-0003] Altieri, M. , and G. Santangelo . 2021. “The Psychological Impact of COVID‐19 Pandemic and Lockdown on Caregivers of People With Dementia.” American Journal of Geriatric Psychiatry 29, no. 1: 27–34. 10.1016/j.jagp.2020.10.009. PMC7577876 33153872

[brb371100-bib-0004] Ayorinde, A. A. , I. Williams , R. Mannion , et al. 2020. “Assessment of Publication Bias and Outcome Reporting Bias in Systematic Reviews of Health Services and Delivery Research: A Meta‐epidemiological Study.” PLoS ONE 15, no. 1: e0227580. 10.1371/JOURNAL.PONE.0227580. 31999702 PMC6992172

[brb371100-bib-0005] Azevedo, L. V. D. S. , I. L. Calandri , A. Slachevsky , et al. 2021. “Impact of Social Isolation on People With Dementia and Their Family Caregivers.” Journal of Alzheimer's Disease 81, no. 2: 607–617. 10.3233/JAD-201580. PMC11185189 33814446

[brb371100-bib-0006] Bakker, E. D. , I. S. Van Maurik , A. Mank , et al. 2022. “Psychosocial Effects of COVID‐19 Measures on (Pre‐)Dementia Patients During Second Lockdown.” Journal of Alzheimer's Disease 86, no. 2: 931–939. 10.3233/JAD-215342. 35034903

[brb371100-bib-0007] Bannon, S. , K. Wang , V. A. Grunberg , B. C. Dickerson , and A. M. Vranceanu . 2021. “Couples' Experiences Managing Young‐onset Dementia Early in the COVID‐19 Pandemic.” The Gerontologist 62, no. 8: 1173–1184. 10.1093/geront/gnab162. PMC9451019 34739072

[brb371100-bib-0008] Barguilla, A. , A. Fernández‐Lebrero , I. Estragués‐Gázquez , et al. 2020. “Effects of COVID‐19 Pandemic Confinement in Patients With Cognitive Impairment.” Frontiers in Neurology 11: 589901. 10.3389/fneur.2020.589901. 33329337 PMC7732426

[brb371100-bib-0009] Baumbusch, J. , H. A. Cooke , K. Seetharaman , A. Khan , and K. B. Khan . 2022. “Exploring the Impacts of COVID‐19 Public Health Measures on Community‐Dwelling People Living With Dementia and Their Family Caregivers: A Longitudinal, Qualitative Study.” Journal of Family Nursing 28, no. 3: 183–194. 10.1177/10748407221100284. 35674313 PMC9280696

[brb371100-bib-0010] Bianchetti, A. , R. Rozzini , L. Bianchetti , F. Coccia , F. Guerini , and M. Trabucchi . 2022. “Dementia Clinical Care in Relation to COVID‐19.” Current Treatment Options in Neurology 24, no. 1: 1–15. 10.1007/S11940-022-00706-7. 35221646 PMC8863507

[brb371100-bib-0011] Bicalho, M. A. C. , M. J. R. Aliberti , P. Delfino‐Pereira , et al. 2024. “Clinical Characteristics and Outcomes of COVID‐19 Patients With Preexisting Dementia: a Large Multicenter Propensity‐matched Brazilian Cohort Study.” BMC Geriatrics 24, no. 1: 25. 10.1186/S12877-023-04494-W. 38182982 PMC10770897

[brb371100-bib-0012] Boutoleau‐Bretonnière, C. , H. Pouclet‐Courtemanche , A. Gillet , et al. 2020. “Impact of Confinement on the Burden of Caregivers of Patients With the Behavioral Variant of Frontotemporal Dementia and Alzheimer Disease During the COVID‐19 Crisis in France.” Dementia and Geriatric Cognitive Disorders Extra 10, no. 3: 127–134. 10.1159/000511416. 34191932 PMC7705930

[brb371100-bib-0013] Burns, J. , A. Movsisyan , J. M. Stratil , et al. 2021. “International Travel‐related Control Measures to Contain the COVID‐19 Pandemic: A Rapid Review.” Cochrane Database of Systematic Reviews (Online) 3, no. 3: CD013717. 10.1002/14651858.CD013717.PUB2. PMC8406796 33763851

[brb371100-bib-0014] Bussè, C. , T. Barnini , M. Zucca , et al. 2022. “Depression, Anxiety and Sleep Alterations in Caregivers of Persons With Dementia After 1‐Year of COVID‐19 Pandemic.” Frontiers in Psychiatry 13: 826371. 10.3389/fpsyt.2022.826371. 35222125 PMC8866969

[brb371100-bib-0015] Canevelli, M. , L. Palmieri , V. Raparelli , et al. 2020. “Prevalence and Clinical Correlates of Dementia Among COVID‐19‐related Deaths in Italy.” Alzheimer's & Dementia 12, no. 1: e12114. 10.1002/DAD2.12114. PMC7666428 33225041

[brb371100-bib-0016] Chen, Y. L. Problems Perceived by People With Dementia and Their Care Partners in Response to the COVID‐19 Control Measures. PROSPERO. https://www.crd.york.ac.uk/PROSPERO/view/CRD42024554701.

[brb371100-bib-0017] Chen, Z. C. , S. Liu , J. H. Gan , et al. 2021. “The Impact of the COVID‐19 Pandemic and Lockdown on Mild Cognitive Impairment, Alzheimer's Disease and Dementia With Lewy Bodies in China: A 1‐Year Follow‐Up Study.” Frontiers in Psychiatry 12: 711658. 34393864 10.3389/fpsyt.2021.711658 PMC8355429

[brb371100-bib-0018] Cipolletta, S. , B. Morandini , and S. C. M. Tomaino . 2023. “Caring for a Person With Dementia During the COVID‐19 Pandemic: A Qualitative Study With family Care‐givers.” Ageing and Society 43, no. 3: 535–555. 10.1017/S0144686X21000696.

[brb371100-bib-0019] Cohen, G. , M. J. Russo , J. A. Campos , and R. F. Allegri . 2020. “COVID‐19 Epidemic in Argentina: Worsening of Behavioral Symptoms in Elderly Subjects With Dementia Living in the Community.” Frontiers in Psychiatry 11: e866. 10.3389/fpsyt.2020.00866. PMC7485090 33005158

[brb371100-bib-0020] Cohen, G. , M. J. Russo , J. A. Campos , and R. F. Allegri . 2020. “Living With Dementia: Increased Level of Caregiver Stress in Times of COVID‐19.” International Psychogeriatrics 32, no. 11: 1377–1381. 10.1017/S1041610220001593. 32729446 PMC7453351

[brb371100-bib-0021] Critical Appraisal Skills Programme . 2024. CASP Checklist: CASP Qualitative Studies Checklist . https://casp-uk.net/casp‐checklists/CASP‐checklist‐qualitative‐2024.pdf.

[brb371100-bib-0022] Daley, S. , N. Farina , L. Hughes , et al. 2022. “Covid‐19 and the Quality of Life of People With Dementia and Their Carers‐The TFD‐C19 Study.” PLoS ONE 17, no. 1: e0262475. 10.1371/journal.pone.0262475. 35045120 PMC8769363

[brb371100-bib-0023] Dwan, K. , C. Gamble , P. R. Williamson , and J. J. Kirkham . 2013. “Systematic Review of the Empirical Evidence of Study Publication Bias and Outcome Reporting Bias—An Updated Review.” PLoS ONE 8, no. 7: e66844. 10.1371/JOURNAL.PONE.0066844. 23861749 PMC3702538

[brb371100-bib-0024] Flemons, K. , G. McGhan , and D. McCaughey . 2022. “Family Caregiving for People Living With Dementia During COVID‐19: A Thematic Analysis.” Journal of Family Nursing 28, no. 3: 219–230. 10.1177/10748407221100553. 35674336 PMC9280697

[brb371100-bib-0025] Gaigher, J. M. , I. B. Lacerda , and M. C. N. Dourado . 2022. “Dementia and Mental Health During the COVID‐19 Pandemic: A Systematic Review.” Frontiers in Psychiatry 13: 879598. 10.3389/FPSYT.2022.879598. 35873228 PMC9301378

[brb371100-bib-0026] Gamble, L. D. , S. Parker , C. Quinn , et al. 2022. “A Comparison of Well‐Being of Carers of People With Dementia and Their Ability to Manage Before and During the COVID‐19 Pandemic: Findings From the IDEAL Study.” Journal of Alzheimer's Disease 88, no. 2: 679–692. 10.3233/JAD-220221. 35634850

[brb371100-bib-0027] Gan, J. , S. Liu , H. Wu , et al. 2021. “The Impact of the COVID‐19 Pandemic on Alzheimer's Disease and Other Dementias.” Frontiers in Psychiatry 12: e703481. 10.3389/fpsyt.2021.703481. PMC8317553 34335338

[brb371100-bib-0028] Geyer, J. , F. Böhm , J. Müller , et al. 2020. “The Life Situation of People With Dementia and family Carers During the Coronavirus Pandemic‐A Qualitative Study.” Pflege 33, no. 4: 189–197. 10.1024/1012-5302/a000750. 32811323

[brb371100-bib-0029] Giebel, C. , K. Hanna , J. Cannon , et al. 2020. “Decision‐making for Receiving Paid Home Care for Dementia in the Time of COVID‐19: A Qualitative Study.” BMC Geriatrics 20, no. 1: 333. 10.1186/s12877-020-01719-0. 32900360 PMC7478902

[brb371100-bib-0030] Giebel, C. , K. Hanna , M. Rajagopal , et al. 2021. “The Potential Dangers of Not Understanding COVID‐19 Public Health Restrictions in Dementia: ‘It's a Groundhog Day—Every Single Day She Does Not Understand Why She Can't Go out for a Walk’.” BMC Public Health [Electronic Resource] 21, no. 1: 762. 10.1186/s12889-021-10815-8. 33879117 PMC8057664

[brb371100-bib-0031] Giebel, C. , K. Hanna , H. Tetlow , et al. 2021. “‘A Piece of Paper Is Not the Same as Having Someone to Talk to’: Accessing Post‐diagnostic Dementia Care Before and Since COVID‐19 and Associated Inequalities.” International Journal for Equity in Health 20, no. 1: 76. 10.1186/s12939-021-01418-1. 33706774 PMC7948657

[brb371100-bib-0032] Giebel, C. , K. Lord , C. Cooper , et al. 2021. “A UK Survey of COVID‐19 Related Social Support Closures and Their Effects on Older People, People With Dementia, and Carers.” International Journal of Geriatric Psychiatry 36, no. 3: 393–402. 10.1002/gps.5434. 32946619 PMC7536967

[brb371100-bib-0033] Giebel, C. , D. Pulford , C. Cooper , et al. 2021. “COVID‐19‐related Social Support Service Closures and Mental Well‐being in Older Adults and Those Affected by Dementia: A UK Longitudinal Survey.” BMJ Open 11, no. 1: e045889. 10.1136/bmjopen-2020-045889. PMC7813330 33455941

[brb371100-bib-0034] Grycuk, E. , Y. Chen , A. Almirall‐Sanchez , et al. 2022. “Care Burden, Loneliness, and Social Isolation in Caregivers of People With Physical and Brain Health Conditions in English‐speaking Regions: Before and During the COVID‐19 Pandemic.” International Journal of Geriatric Psychiatry 37, no. 6: 453–462. 10.1002/gps.5734. PMC9324775 35574817

[brb371100-bib-0035] Hanafy, S. , A. Colantonio , T. Mollayeva , S. Munce , and S. Lindsay . 2023. “Employment and Accommodation Needs and the Effect of COVID‐19 on Men and Women With Traumatic Brain Injury.” Work (Reading, Mass) 75, no. 1: 41–58. 10.3233/WOR-220437. 36591690

[brb371100-bib-0036] Hariyanto, T. I. , C. Putri , J. Arisa , R. F. V. Situmeang , and A. Kurniawan . 2021. “Dementia and Outcomes From Coronavirus Disease 2019 (COVID‐19) Pneumonia: A Systematic Review and Meta‐Analysis.” Archives of Gerontology and Geriatrics 93: 104299. 10.1016/J.ARCHGER.2020.104299. 33285424 PMC7674980

[brb371100-bib-0037] Hashimoto, M. , M. Suzuki , M. Hotta , et al. 2020. “The Influence of the COVID‐19 Outbreak on the Lifestyle of Older Patients With Dementia or Mild Cognitive Impairment Who Live Alone.” Frontiers in Psychiatry 11: 570580. 10.3389/fpsyt.2020.570580. 33192695 PMC7661779

[brb371100-bib-0038] Helvaci Yilmaz, N. , B. Polat , A. Ermis , and L. Hanoglu . 2021. “Clinical Deterioration of Alzheimer's Disease Patients During the Covid‐19 Pandemic and Caregiver Burden.” Journal of Experimental and Clinical Medicine 38, no. 3: 255–259. 10.52142/omujecm.38.3.9.

[brb371100-bib-0039] Henry, A. , M. Katsoulis , S. Masi , et al. 2019. “The Relationship Between Sleep Duration, Cognition and Dementia: A Mendelian Randomization Study.” International Journal of Epidemiology 48, no. 3: 849–860. 10.1093/IJE/DYZ071. 31062029 PMC6659373

[brb371100-bib-0040] Hicks, B. , S. Read , B. Hu , et al. 2022. “A Cohort Study of the Impact of COVID‐19 on the Quality of Life of People Newly Diagnosed With Dementia and Their family Carers.” Alzheimers and Dementia‐Translational Research and Clinical Interventions 8, no. 1: e12236. 10.1002/trc2.12236 PMC9060551 35509503

[brb371100-bib-0041] Ismail, I. I. , W. A. Kamel , and J. Y. Al‐Hashel . 2021. “Association of COVID‐19 Pandemic and Rate of Cognitive Decline in Patients With Dementia and Mild Cognitive Impairment: A Cross‐Sectional Study.” Gerontology & Geriatric Medicine 7: 23337214211005223. 33816709 10.1177/23337214211005223 PMC7995295

[brb371100-bib-0042] Jones, A. , L. C. Maclagan , C. Schumacher , et al. 2021. “Impact of the COVID‐19 Pandemic on Home Care Services Among Community‐Dwelling Adults With Dementia.” Journal of the American Medical Directors Association 22: 2258–2262.e1. 10.1016/j.jamda.2021.08.031. 34571041 PMC8422852

[brb371100-bib-0043] Kennedy, P. , C. Rogan , D. Higgins , et al. 2024. “Changes and Interruptions During COVID‐19: Caregivers of People With Brain Health Challenges‐A Qualitative Analysis.” Frontiers in Dementia 3: e1360112. 10.3389/FRDEM.2024.1360112. PMC11285620 39081614

[brb371100-bib-0044] Kostyál, L. , Z. Széman , V. Almási , et al. 2021. “Impact of the COVID‐19 Pandemic on Family Carers of Older People Living With Dementia in Italy and Hungary.” Sustainability 13, no. 13: 7107. 10.3390/su13137107.

[brb371100-bib-0045] Kostyál, L. Á. , Z. Széman , V. E. Almási , et al. 2022. “The Impact of COVID‐19 on the Health and Experience of the Carers of Family Members Living With Dementia: An Italian‐Hungarian Comparative Study.” International Journal of Environmental Research and Public Health 19, no. 9: 5329. 10.3390/ijerph19095329. 35564723 PMC9104228

[brb371100-bib-0046] Kuroda, Y. , T. Sugimoto , N. Matsumoto , et al. 2022. “Prevalence of Behavioral and Psychological Symptoms in Patients With Cognitive Decline Before and During the COVID‐19 Pandemic.” Frontiers in Psychiatry 13: e839683. 10.3389/fpsyt.2022.839683. PMC8934776 35321225

[brb371100-bib-0047] Lara, B. , A. Carnes , F. Dakterzada , I. Benitez , and G. Piñol‐Ripoll . 2020. “Neuropsychiatric Symptoms and Quality of Life in Spanish Patients With Alzheimer's Disease During the COVID‐19 Lockdown.” European Journal of Neurology 27, no. 9: 1744–1747. 10.1111/ene.14339. 32449791 PMC7283827

[brb371100-bib-0048] Lee, Y. , L. M. W. Lui , D. Chen‐Li , et al. 2021. “Government Response Moderates the Mental Health Impact of COVID‐19: A Systematic Review and Meta‐Analysis of Depression Outcomes Across Countries.” Journal of Affective Disorders 290: 364–377. 10.1016/J.JAD.2021.04.050. 34052584 PMC8159271

[brb371100-bib-0049] Lion, K. M. , W. Moyle , M. Cations , et al. 2022. “How Did the COVID‐19 Restrictions Impact People Living With Dementia and Their Informal Carers Within Community and Residential Aged Care Settings in Australia? A Qualitative Study.” Journal of Family Nursing 28, no. 3: 205–218. 10.1177/10748407221101638. 35674356

[brb371100-bib-0050] Livingston, G. , H. Rostamipour , P. Gallagher , et al. 2020. “Prevalence, Management, and Outcomes of SARS‐CoV‐2 Infections in Older People and Those With Dementia in Mental Health Wards in London, UK: A Retrospective Observational Study.” Lancet Psychiatry 7, no. 12: 1054–1063. 10.1016/S2215-0366(20)30434-X. 33031760 PMC7535621

[brb371100-bib-0051] Llibre‐Rodriguez, J. J. , I. P. Santiesteban , L. Noriega‐Fernandez , et al. 2021. “Overburden and Correlates Among Caregivers of People With Dementia During the COVID‐19 Pandemic.” [In Spanish] Revista Habanera de Ciencias Medicas Revista Habanera De Ciencias Medicas 20, no. 4: e3944.

[brb371100-bib-0052] Maćkowiak, M. , A. Senczyszyn , K. Lion , et al. 2021. “The Experiences of People With Dementia and Informal Carers Related to the Closure of Social and Medical Services in Poland During the COVID‐19 Pandemic‐A Qualitative Study.” Healthcare 9, no. 12: 1677. 10.3390/healthcare9121677. 34946403 PMC8702134

[brb371100-bib-0053] Maclagan, L. C. , X. Wang , A. Emdin , et al. 2022. “Visits to the Emergency Department by Community‐dwelling People With Dementia During the First 2 Waves of the COVID‐19 Pandemic in Ontario: A Repeated Cross‐sectional Analysis.” CMAJ Open 10, no. 3: E610–E621. 10.9778/cmajo.20210301. PMC9262349 35790227

[brb371100-bib-0054] Maggio, M. G. , G. La Rosa , P. Calatozzo , et al. 2021. “How COVID‐19 Has Affected Caregivers' Burden of Patients With Dementia: An Exploratory Study Focusing on Coping Strategies and Quality of Life During the Lockdown.” Journal of Clinical Medicine 10, no. 24: 5953. 10.3390/jcm10245953. 34945251 PMC8704515

[brb371100-bib-0055] Magklara, K. , and M. Kyriakopoulos . 2023. “The Impact of the COVID‐19 Pandemic on Children and Young People.” Psychiatriki 34, no. 4: 265–268. 10.22365/JPSYCH.2023.024. 37982248

[brb371100-bib-0056] Manca, R. , M. De Marco , A. Colston , et al. 2021. “The Impact of Social Isolation due to the COVID‐19 Pandemic on Patients With Dementia and Caregivers.” Acta Neuropsychiatrica 33, no. 1: 50–57. 10.1017/neu.2022.12 35369891

[brb371100-bib-0057] Manini, A. , M. Brambilla , L. Maggiore , S. Pomati , and L. Pantoni . 2021. “The Impact of Lockdown During SARS‐CoV‐2 Outbreak on Behavioral and Psychological Symptoms of Dementia.” Neurological Sciences 42, no. 3: 825–833. 10.1007/s10072-020-05035-8. 33442845 PMC7806279

[brb371100-bib-0058] Mohammadian, F. , M. Rezaee , A. Kalantar , N. Mohebbi , and M. Motamed . 2022. “Relationship Between Psychological Impacts of COVID‐19 and Loneliness in Patients With Dementia: A Cross‐Sectional Study From Iran.” Frontiers in Psychiatry 13: e814676. 10.3389/fpsyt.2022.814676. PMC9019135 35463502

[brb371100-bib-0059] Moretti, R. , P. Caruso , M. Giuffré , and C. Tiribelli . 2021. “COVID‐19 Lockdown Effect on Not Institutionalized Patients With Dementia and Caregivers.” Healthcare 9, no. 7: 893. 10.3390/healthcare9070893. 34356269 PMC8303803

[brb371100-bib-0060] Morkavuk, G. , A. Demirkol , G. E. Berber , et al. 2021. “Comparison of Dementia Patients Admission Rates and Dementia Characteristics before and during the COVID‐19 Pandemic.” Cureus 13, no. 11: e19934. 10.7759/CUREUS.19934. 34976529 PMC8712250

[brb371100-bib-0061] National Institutes of Health . 2013. Study Quality Assessment Tools .

[brb371100-bib-0062] Oliver, S. , K. Alexander , S. G. Bennett , et al. 2022. “Experiences of Black American Dementia Caregivers During the COVID‐19 Pandemic.” Journal of Family Nursing 28, no. 3: 195–204. 10.1177/10748407221102465. 35674329 PMC9280120

[brb371100-bib-0063] Oluwasegun Ayenigbara, I. 2022. “Mental Health Amid COVID‐19 Pandemic: Appropriate Coping Strategies.” Psychiatria Danubina 34, no. 2: 325–333. 10.24869/PSYD.2022.325. 35772154

[brb371100-bib-0064] Page, M. J. , J. E. McKenzie , P. M. Bossuyt , et al. 2021. “The PRISMA 2020 Statement: An Updated Guideline for Reporting Systematic Reviews.” BMJ 372: n71. 10.1136/BMJ.N71. 33782057 PMC8005924

[brb371100-bib-0065] Paolini, S. , M. Devita , O. M. Epifania , et al. 2021. “Perception of Stress and Cognitive Efficiency in Older Adults With Mild and Moderate Dementia During the COVID‐19‐related Lockdown.” Journal of Psychosomatic Research 149: 110584. 10.1016/j.jpsychores.2021.110584. 34340137 PMC9749757

[brb371100-bib-0066] Penteado, C. T. , J. C. Loureiro , M. V. Pais , et al. 2020. “Mental Health Status of Psychogeriatric Patients During the 2019 New Coronavirus Disease (COVID‐19) Pandemic and Effects on Caregiver Burden.” Frontiers in Psychiatry 11: e578672. 10.3389/fpsyt.2020.578672. PMC7704440 33312138

[brb371100-bib-0067] Perach, R. , S. Read , B. Hicks , et al. 2022. “Predictors of Loneliness During the Covid‐19 Pandemic in People With Dementia and Their Carers in England: Findings From the DETERMIND‐C19 Study.” Aging and Mental Health 27, no. 3: 521–532. 10.1080/13607863.2022.2080179. 35658781

[brb371100-bib-0068] Pickering, C. E. Z. , C. D. Maxwell , M. Yefimova , D. Wang , F. Puga , and T. Sullivan . 2022. “Early Stages of Covid‐19 Pandemic Had no Discernable Impact on Risk of Elder Abuse and Neglect Among Dementia family Caregivers: A Daily Diary Study.” Journal of Family Violence 11: 1–11. 10.1007/s10896-022-00392-8 PMC9095055 35578604

[brb371100-bib-0069] Prather, A. A. , N. Vogelzangs , and B. Penninx . 2015. “Sleep Duration, Insomnia, and Markers of Systemic Inflammation: Results From the Netherlands Study of Depression and Anxiety (NESDA).” Journal of Psychiatric Research 60: 95–102. 10.1016/J.JPSYCHIRES.2014.09.018. 25307717 PMC4250403

[brb371100-bib-0070] Quinn, C. , L. D. Gamble , S. Parker , et al. 2022. “Impact of COVID‐19 on Carers of People With Dementia in the Community: Findings From the British IDEAL Cohort.” International Journal of Geriatric Psychiatry 37, no. 5: 1–11. 10.1002/gps.5708. PMC9087398 35394090

[brb371100-bib-0071] Rainero, I. , A. C. Bruni , C. Marra , et al. 2021. “The Impact of COVID‐19 Quarantine on Patients With Dementia and Family Caregivers: A Nation‐Wide Survey.” Frontiers in Aging Neuroscience 12: 625781. 10.3389/fnagi.2020.625781. 33536898 PMC7849158

[brb371100-bib-0072] Roach, P. , A. Zwiers , E. Cox , et al. 2021. “Understanding the Impact of the COVID‐19 Pandemic on Well‐being and Virtual Care for People Living With Dementia and Care Partners Living in the Community.” Dementia 20, no. 6: 2007–2023. 10.1177/1471301220977639. 33381996 PMC7952494

[brb371100-bib-0073] Rusowicz, J. , K. Pezdek , and J. Szczepańska‐Gieracha . 2021. “Needs of Alzheimer's Charges' Caregivers in Poland in the Covid‐19 Pandemic‐An Observational Study.” International Journal of Environmental Research and Public Health 18, no. 9: 4493. 10.3390/ijerph18094493. 33922673 PMC8122957

[brb371100-bib-0074] Russo, M. J. , G. Cohen , J. Campos , and R. F. Allegri . 2021. “COVID‐19 and Older Adults With Cognitive Impairment: How Social Isolation Affects the Disease?” Neurologia Argentina 13, no. 3: 159–169. 10.1016/j.neuarg.2021.06.003.

[brb371100-bib-0075] Sabatini, S. , H. Q. Bennett , A. Martyr , et al. 2022. “Minimal Impact of COVID‐19 Pandemic on the Mental Health and Wellbeing of People Living With Dementia: Analysis of Matched Longitudinal Data From the IDEAL Study.” Frontiers in Psychiatry 13: e849808. 10.3389/fpsyt.2022.849808. PMC8965515 35370851

[brb371100-bib-0076] Salanti, G. , N. Peter , T. Tonia , et al. 2022. “The Impact of the COVID‐19 Pandemic and Associated Control Measures on the Mental Health of the General Population: A Systematic Review and Dose‐Response Meta‐analysis.” Annals of Internal Medicine 175, no. 11: 1560–1571. 10.7326/M22-1507. 36252247 PMC9579966

[brb371100-bib-0077] Sánchez‐Teruel, D. , M. A. Robles‐Bello , M. Sarhani‐Robles , and A. Sarhani‐Robles . 2022. “Exploring Resilience and Well‐being of family Caregivers of People With Dementia Exposed to Mandatory Social Isolation by COVID‐19.” Dementia 21, no. 2: 410–425. 10.1177/14713012211042187. 34517732 PMC8818476

[brb371100-bib-0078] Sant'Ana, T. T. , S. Hanafy , E. Fuller‐Thomson , et al. 2024. “A PROGRESS‐driven Approach to Cognitive Outcomes After Traumatic Brain Injury: A Study Protocol for Advancing Equity, Diversity, and Inclusion Through Knowledge Synthesis and Mobilization.” PLoS ONE 19, no. 7: e0307418. 10.1371/JOURNAL.PONE.0307418. 39037993 PMC11262676

[brb371100-bib-0079] Scottish Intercollegiate Guidelines Network . Methodology Checklist .

[brb371100-bib-0080] Slavin, R. E. 1995. “Best Evidence Synthesis: An Intelligent Alternative to Meta‐Analysis.” Journal of Clinical Epidemiology 48, no. 1: 9–18. 10.1016/0895-4356(94)00097-A. 7853053

[brb371100-bib-0081] Sriram, V. , C. Jenkinson , and M. Peters . 2021. “Impact of COVID‐19 Restrictions on Carers of Persons With Dementia in the UK: A Qualitative Study.” Age and Ageing 50, no. 6: 1876–1885. 10.1093/ageing/afab156. 34224555 PMC8384409

[brb371100-bib-0082] Stratil, J. M. , R. L. Biallas , J. Burns , et al. 2021. “Non‐pharmacological Measures Implemented in the Setting of Long‐Term Care Facilities to Prevent SARS‐CoV‐2 Infections and Their Consequences: A Rapid Review.” Cochrane Database of Systematic Reviews (Online) 9, no. 9: CDO15085. 10.1002/14651858.CD015085.PUB2. PMC8442144 34523727

[brb371100-bib-0083] Stubbs, M. , I. Govia , J. N. Robinson , R. Amour , and E. Freeman . 2021. “The Experiences of Caregivers of Persons Living With Dementia in Jamaica During COVID‐19.” Gerontology and Geriatric Medicine 7: 23337214211043384. 10.1177/23337214211043384. 34595330 PMC8477703

[brb371100-bib-0084] Suárez‐González, A. , J. Rajagopalan , G. Livingston , and S. Alladi . 2021. “The Effect of COVID‐19 Isolation Measures on the Cognition and Mental Health of People Living With Dementia: A Rapid Systematic Review of One Year of Quantitative Evidence.” EClinicalMedicine 39: 101047. 10.1016/J.ECLINM.2021.101047. 34386758 PMC8342894

[brb371100-bib-0085] Talbot, C. V. , and P. Briggs . 2021. “‘Getting Back to Normality Seems as Big of a Step as Going Into Lockdown’: The Impact of the COVID‐19 Pandemic on People With Early to Middle Stage Dementia.” Age and Ageing 50, no. 3: 657–663. 10.1093/ageing/afab012. 33481988 PMC7929391

[brb371100-bib-0086] Talic, S. , S. Shah , H. Wild , et al. 2021. “Effectiveness of Public Health Measures in Reducing the Incidence of Covid‐19, SARS‐CoV‐2 Transmission, and Covid‐19 Mortality: Systematic Review and Meta‐Analysis.” BMJ 375: n2527. 10.1136/BMJ-2021-068302. 34789505 PMC9423125

[brb371100-bib-0087] Tavares‐Júnior, J. W. L. , A. C. C. De Souza , J. W. P. Borges , et al. 2022. “COVID‐19 Associated Cognitive Impairment: A Systematic Review.” Cortex; A Journal Devoted to the Study of the Nervous System and Behavior 152: 77–97. 10.1016/J.CORTEX.2022.04.006. 35537236 PMC9014565

[brb371100-bib-0088] Theurer, C. , D. Rother , K. Pfeiffer , and G. Wilz . 2022. “Burden Experienced by Caregiving Relatives During the Corona Pandemic.” Zeitschrift Fur Gerontologie Und Geriatrie 55, no. 2: 136–142. 10.1007/s00391-022-02026-6. 35166934 PMC8852872

[brb371100-bib-0089] Tondo, G. , B. Sarasso , P. Serra , F. Tesser , and C. Comi . 2021. “The Impact of the COVID‐19 Pandemic on the Cognition of People With Dementia.” International Journal of Environmental Research and Public Health 18, no. 8: 4285. 10.3390/ijerph18084285. 33919491 PMC8073614

[brb371100-bib-0090] Tsapanou, A. , J. D. Papatriantafyllou , K. Yiannopoulou , et al. 2021. “The Impact of COVID‐19 Pandemic on People With Mild Cognitive Impairment/Dementia and on Their Caregivers.” International Journal of Geriatric Psychiatry 36, no. 4: 583–587. 10.1002/gps.5457. 33166418

[brb371100-bib-0091] Tuijt, R. , R. Frost , J. Wilcock , et al. 2021. “Life Under Lockdown and Social Restrictions—The Experiences of People Living With Dementia and Their Carers During the COVID‐19 Pandemic in England.” BMC Geriatrics 21, no. 1: 301. 10.1186/s12877-021-02257-z. 33971847 PMC8107803

[brb371100-bib-0092] Vaitheswaran, S. , M. Lakshminarayanan , V. Ramanujam , S. Sargunan , and S. Venkatesan . 2020. “Experiences and Needs of Caregivers of Persons With Dementia in India During the COVID‐19 Pandemic‐A Qualitative.” American Journal of Geriatric Psychiatry 28, no. 11: 1185–1194. 10.1016/j.jagp.2020.06.026. PMC7340037 32736918

[brb371100-bib-0093] van Maurik, I. S. , E. D. Bakker , S. van den Buuse , et al. 2020. “Psychosocial Effects of Corona Measures on Patients With Dementia, Mild Cognitive Impairment and Subjective Cognitive Decline.” Frontiers in Psychiatry 11: 585686. 33192733 10.3389/fpsyt.2020.585686 PMC7649118

[brb371100-bib-0094] Vislapuu, M. , R. C. Angeles , L. I. Berge , E. Kjerstad , M. H. Gedde , and B. S. Husebo . 2021. “The Consequences of COVID‐19 Lockdown for Formal and Informal Resource Utilization Among Home‐dwelling People With Dementia: Results From the Prospective PAN.DEM Study.” BMC Health Services Research [Electronic Resource] 21, no. 1: 1003. 10.1186/s12913-021-07041-8. 34551783 PMC8457031

[brb371100-bib-0095] Wang, Z. , J. Whittington , H. Y. Yuan , H. Miao , H. Tian , and N. C. Stenseth . 2021. “Evaluating the Effectiveness of Control Measures in Multiple Regions During the Early Phase of the COVID‐19 Pandemic in 2020.” Biosafety and Health 3, no. 5: 264–275. 10.1016/J.BSHEAL.2021.09.002. 34541485 PMC8436421

[brb371100-bib-0096] Wei, G. , J. Diehl‐Schmid , J. A. Matias‐Guiu , et al. 2022. “The Effects of the COVID‐19 Pandemic on Neuropsychiatric Symptoms in Dementia and Carer Mental Health: An International Multicentre Study.” Scientific Reports 12, no. 1: 2418. 10.1038/s41598-022-05687-w. 35165292 PMC8844310

[brb371100-bib-0097] Werner, P. , A. Tur‐Sinai , and H. AboJabel . 2021. “Examining Dementia Family Caregivers' Forgone Care for General Practitioners and Medical Specialists During a COVID‐19 Lockdown.” International Journal of Environmental Research and Public Health 18, no. 7: 3688. 10.3390/ijerph18073688. 33916152 PMC8036927

[brb371100-bib-0098] West, E. , P. Nair , Y. Barrado‐Martin , et al. 2021. “Exploration of the Impact of the COVID‐19 Pandemic on People With Dementia and Carers From Black and Minority Ethnic Groups.” BMJ Open 11, no. 5: e050066. 10.1136/bmjopen-2021-050066. PMC8136797 34006561

[brb371100-bib-0099] Yuan, S. , W. Zhang , W. Lü , et al. 2021. “The Psychological Impact on Patients With Memory Disorders and Their Caregivers During COVID‐19.” Aging Clinical and Experimental Research 33, no. 8: 2317–2325. 10.1007/s40520-021-01911-1. 34159534 PMC8219516

[brb371100-bib-0100] Yuan, S. , W. Zhang , Q. Yao , et al. 2022. “The Neuropsychiatric Changes after COVID‐19 Quarantine in Patients With Cognitive Impairment and Their Caregivers in Chongqing, China: A Cohort Study.” Frontiers in Aging Neuroscience 14: 1–9. 10.3389/fnagi.2021.762907 PMC8868570 35221981

[brb371100-bib-0101] Zhang, Y. , E. W. J. Lee , and W. P. Teo . 2023. “Health‐Seeking Behavior and Its Associated Technology Use: Interview Study Among Community‐Dwelling Older Adults.” JMIR Aging 6: e43709. 10.2196/43709. 36996003 PMC10196894

