# Supplementary material for: The Global Impact of COVID‐19 Control Measures on People With Dementia Living at Home and Their Carers: A Systematic Review of Quantitative and Qualitative Research Across 27 Countries
Source: Brain Behav. 2025 Nov 24;15(11):e71100. doi: 10.1002/brb3.71100 (PMC12641281; doi:10.1002/brb3.71100)
Supplement: Supplementary file 3 — Supplementary Materials: brb371100‐sup‐0003‐SuppMat.docx [file BRB3-15-e71100-s003.docx]

**Supplementary File 3. Quality Assessment for Quantitative Studies (NIH Quality Assessment Tool & QUIPS Used)**

**Terms Used:** CD, cannot determine; NA, not applicable; NR, not reported

**Notes:** Items 1 and 2 rated as Yes, if at minimum, aim(s) and objective (s) stated and inclusion criteria specified in the manuscript. Item 5 is rated as Yes, if any of the questions was answered as Yes. Item 7 was rated as CD for all studies- please refer to the justification above. Items 9 and 11 rated as Yes, if at minimum, measures were described. Item 14 required the following variables, at least, to be positioned as confounding variables-age, sex, severity/type of dementia or level of impairment to satisfy rating Yes.

**Summary Rating:**

In addition to crude calculation of total number of biases present out of total number of possible, each study quality was summarized qualitatively into three groups: i) “++” when all or most of the quality were fulfilled (i.e. allowing one “CD” or “NR” based six potential sources of bias (i.e. study participation, design and attrition, associated factors, outcome measurements, confounding variables, and analyses).); ii) “+” when half items of criteria were fulfilled; iii) “-” when less than half of items of criteria were fulfilled.

| **QUIPS Six Potential Sources of Bias** | | **Assessment Criteria** | |
| --- | --- | --- | --- |
| 1. | Study Design | 1.  10.  12. | Was the research question or objective in this paper clearly stated?  Was the exposure(s) assessed more than once over time?  Were the outcome assessors blinded to the exposure status of participants? |
| 2. | Study Participation | 2.  3.  4. | Was the study population clearly specified and defined?  Was the participation rate of eligible persons at least 50%?  Were all the subjects selected or recruited from the same or similar populations (including the same time period)? Were inclusion and exclusion criteria for being in the study prespecified and applied uniformly to all participants? |
| 3. | Study Attrition | 13. | Was loss to follow-up after baseline 20% or less? |
| 4. | Associated Factors | 9. | Were the exposure measures (independent variables) clearly defined, valid, reliable, and implemented consistently across all study participants? |
| 5. | Outcome Measures/ Confounding Account | 11.  14. | Were the outcome measures (dependent variables) clearly defined, valid, reliable, and implemented consistently across all study participants?  Were key potential confounding variables measured and adjusted statistically for their impact on the relationship between exposure(s) and outcome(s)? |
| 6. | Analysis | 5.  6.  7.  8. | Was a sample size justification, power description, or variance and effect estimates provided?  For the analyses in this paper, were the exposure(s) of interest measured prior to the outcome(s) being measured?  Was the timeframe sufficient so that one could reasonably expect to see an association between exposure and outcome if it existed?  For exposures that can vary in amount or level, did the study examine different levels of the exposure as related to the outcome (e.g., categories of exposure, or exposure measured as continuous variable)? |

**Quality Assessment for Quantitative Studies**

| # | Study first author, year | 1 | 2 | 3 | 4 | 5 | 6 | 7 | 8 | 9 | 10 | 11 | 12 | 13 | 14 | Score | Rating |
| --- | --- | --- | --- | --- | --- | --- | --- | --- | --- | --- | --- | --- | --- | --- | --- | --- | --- |
| 1. | Alexopoulos, 2021 | Yes | Yes | NR | Yes | No | NA | CD | NA | Yes | NA | Yes | No | NA | No | 5/9 | + |
| 2. | Altieri, 2021 | Yes | No | NR | CD | No | NA | CD | NA | Yes | NA | Yes | No | NA | No | 3/9 | _ |
| 3. | Azevedo, 2021 | Yes | Yes | NR | Yes | No | NA | CD | NA | CD | NA | CD | No | NA | No | 3/9 | _ |
| 4. | Bakker, 2022 | Yes | Yes | No | No | Yes | NA | CD | NA | Yes | NA | Yes | No | NA | Yes | 6/9 | + |
| 5. | Barguilla, 2020 | Yes | Yes | NR | Yes | No | NA | CD | NA | Yes | NA | Yes | No | NA | No | 5/9 | + |
| 6. | Boutoleau- Bretonniere, 2022 | Yes | Yes | NR | Yes | No | NA | CD | NA | Yes | NA | Yes | No | NA | No | 5/9 | + |
| 7. | Busse, 2022 | Yes | Yes | CD | No | Yes | No | CD | No | Yes | Yes | Yes | No | Yes | No | 7/14 | + |
| 8. | Chen, 2021 | Yes | Yes | NA | Yes | No | NA | CD | NA | Yes | NA | Yes | No | NA | Yes | 6/9 | + |
| 9. | Cohen, 2020a | Yes | Yes | NR | Yes | No | NA | CD | NA | CD | NA | CD | No | NA | No | 3/9 | _ |
| 10. | Cohen, 2020b | Yes | Yes | NR | No | No | NA | CD | NA | Yes | NA | Yes | No | NA | No | 4/9 | - |
| 11. | Daley, 2022 | Yes | Yes | NR | Yes | No | Yes | CD | Yes | CD | Yes | Yes | No | NR | No | 7/14 | + |
| 12. | Llibre- Rodriguez, 2021 | Yes | Yes | No | No | No | NA | CD | NA | Yes | NA | Yes | No | NA | No | 4/9 | - |
| 13. | Gamble, 2022 | Yes | Yes | No | Yes | Yes | Yes | CD | Yes | Yes | Yes | Yes | No | No | Yes | 10/13 | + |
| 14 | Gan, 2021 | Yes | Yes | NA | No | No | NA | CD | NA | Yes | NA | Yes | No | NA | No | 5/8 | + |
| 15. | Giebel, 2021 | Yes | Yes | NR | CD | Yes | No | CD | No | CD | Yes | Yes | No | No | No | 5/14 | _ |
| 16. | Giebel, 2022 | Yes | Yes | NR | No | No | NA | CD | NA | Yes | NA | CD* | No | NA | No | 3/9 | _ |
| 17. | Grycuk, 2022 | Yes | Yes | NR | CD | Yes | NA | CD | NA | Yes | NA | Yes | No | NA | No | 5/9 | + |
| 18. | Hashimoto, 2020 | Yes | Yes | NR | No | No | NA | CD | NA | Yes | NA | Yes | No | NA | No | 4/9 | - |
| 19. | Hicks, 2021 | Yes | Yes | NR | No | Yes | NA | CD | Yes | Yes | NA | Yes | No | No | Yes | 7/11 | + |
| 20. | Helvaci Yilmaz, 2021 | Yes | Yes | NR | Yes | No | NA | CD | NA | Yes | NA | Yes | No | NA | No | 5/9 | + |
| 21. | Ismail, 2021 | Yes | Yes | NR | Yes | No | NA | CD | No | Yes | NA | Yes | No | NR | Yes | 6/11 | + |
| 22. | Jones., 2021 | Yes | Yes | Yes | NR | Yes | Yes | CD | Yes | Yes | Yes | Yes | No | Yes | No | 10/14 | + |
| 23. | Kostyal, 2022 | Yes | Yes | No | Yes | No | NA | CD | NA | Yes | NA | No | No | NA | No | 4/9 | - |
| 24. | Kuroda, 2022 | Yes | Yes | NR | Yes | Yes | NA | CD | NA | Yes | NA | Yes | No | NA | Yes | 7/9 | + |
| 25. | Lara, 2020 | Yes | Yes | NR | Yes | No | Yes | CD | No | Yes | No | Yes | No | Yes | No | 7/14 | + |
| 26. | Maclagan, 2022 | Yes | Yes | NA | No | Yes | Yes | CD | NA | Yes | NA | Yes | No | NA | No | 6/9 | + |
| 27. | Maggio, 2021 | Yes | Yes | Yes | Yes | No | NA | CD | NA | Yes | NA | Yes | No | NA | No | 6/9 | + |
| 28. | Manca, 2022 | Yes | Yes | NR | No | Yes | NA | CD | NA | Yes | NA | Yes | No | NA | No | 5/9 | + |
| 29. | Manini, 2021 | Yes | Yes | NR | CD | Yes | NA | CD | NA | Yes | No | Yes | No | NA | Yes | 6/9 | + |
| 30. | Mohammadian, 2022 | Yes | Yes | NR | No | Yes | NA | CD | NA | Yes | NA | Yes | No | NA | No | 5/9 | + |
| 31. | Moretti, 2021 | Yes | Yes | Yes | Yes | No | NA | CD | No | Yes | NA | Yes | No | NA | Yes | 7/10 | + |
| 32. | Morkavuk, 2021 | Yes | Yes | NR | No | No | NA | CD | NA | Yes | NA | Yes | No | NA | No | 4/9 | - |
| 33. | Paolini, 2021 | Yes | Yes | NR | Yes | No | Yes | CD | Yes | Yes | Yes | Yes | No | No | No | 8/14 | + |
| 34. | Penteado, 2020 | Yes | Yes | No | Yes | Yes | NA | CD | NA | Yes | NA | Yes | No | NA | No | 6/9 | + |
| 35. | Perach, 2022 | Yes | Yes | NR | Yes | Yes | Yes | CD | CD | Yes | Yes | Yes | No | Yes | No | 9/14 | + |
| 36. | Pickering, 2022 | Yes | Yes | NR | CD | No | Yes | CD | No | Yes | Yes | Yes | No | No | No | 6/14 | _ |
| 37. | Quinn, 2022 | Yes | Yes | No | No | Yes | NA | CD | NA | Yes | NA | Yes | No | NA | No | 5/9 | + |
| 38. | Rainero, 2021 | Yes | Yes | NR | Yes | No | NA | CD | NA | Yes | NA | Yes | No | NA | Yes | 5/9 | + |
| 39. | Rusowicz, 2021 | Yes | Yes | NR | CD | No | NA | CD | NA | Yes | NA | Yes | No | NA | No | 4/9 | _ |
| 40. | Russo, 2021 | Yes | Yes | NR | No | No | NA | CD | NA | Yes | NA | Yes | No | NA | No | 4/9 | - |
| 41. | Sabatini, 2022 | Yes | Yes | CD | No | Yes | NA | CD | NA | Yes | NA | Yes | No | NA | Yes | 6/10 | + |
| 42. | Sanchez- Teruel al, 2022 | Yes | Yes | CD | Yes | Yes | NA | CD | NA | Yes | NA | Yes | No | NA | Yes | 7/9 | + |
| 43. | Theurer, 2022 | Yes | Yes | Yes | Yes | Yes | NA | CD | NA | Yes | NA | Yes | No | NA | No | 7/9 | + |
| 44. | Tondo, 2021 | Yes | Yes | No | Yes | Yes | Yes | CD | No | Yes | Yes | Yes | No | No | No | 8/14 | + |
| 45. | Tsapanou, 2021 | Yes | Yes | NR | CD | Yes | NA | CD | NA | CD | NA | CD | No | NA | No | 3/9 | _ |
| 46. | Vislapuu, 2021 | Yes | Yes | No | CD | Yes | Yes | CD | No | Yes | No | Yes | No | Yes | No | 7/14 | + |
| 47. | van Maurik 2020 | Yes | Yes | No | No | Yes | NA | CD | NA | Yes | NA | No | No | NA | No | 4/9 | _ |
| 48. | Wei, 2022 | Yes | Yes | CD | CD | No | NA | CD | NA | Yes | NA | Yes | No | NA | Yes | 5/9 | + |
| 49. | Werner, 2021 | Yes | Yes | NR | Yes | No | NA | CD | NA | Yes | NA | Yes | No | NA | No | 5/9 | + |
| 50. | Yuan, 2021 | Yes | Yes | No | Yes | No | NA | CD | NA | Yes | NA | Yes | No | NA | No | 5/9 | + |
| 51. | Yuan, 2022 | Yes | Yes | No | Yes | Yes | No | CD | No | Yes | Yes | Yes | No | No | No | 7/13 | + |
